# Transcriptional and functional effects of lithium in bipolar disorder iPSC-derived cortical spheroids

**DOI:** 10.1038/s41380-023-01944-0

**Published:** 2023-01-18

**Authors:** Jordi Requena Osete, Ibrahim A. Akkouh, Oleksandr Ievglevskyi, Matthieu Vandenberghe, Denis Reis de Assis, Thor Ueland, Elena Kondratskaya, Børge Holen, Attila Szabo, Timothy Hughes, Olav B. Smeland, Vidar Martin Steen, Ole A. Andreassen, Srdjan Djurovic

**Affiliations:** 1 Department of Medical Genetics, Oslo University Hospital, Oslo, Norway; 2 NORMENT, Institute of Clinical Medicine, University of Oslo, and Division of Mental Health and Addiction, Oslo University Hospital, Oslo, Norway; 3 Research Institute of Internal Medicine, Oslo University Hospital Rikshospitalet, Oslo, Norway; 4 NORMENT, Department of Clinical Science, University of Bergen, Bergen, Norway

**Keywords:** Stem cells, Bipolar disorder, Genetics, Molecular biology, Cell biology

## Abstract

Lithium (Li) is recommended for long-term treatment of bipolar disorder (BD). However, its mechanism of action is still poorly understood. Induced pluripotent stem cell (iPSC)-derived brain organoids have emerged as a powerful tool for modeling BD-related disease mechanisms. We studied the effects of 1 mM Li treatment for 1 month in iPSC-derived human cortical spheroids (hCS) from 10 healthy controls (CTRL) and 11 BD patients (6 Li-responders, Li-R, and 5 Li non-treated, Li-N). At day 180 of differentiation, BD hCS showed smaller size, reduced proportion of neurons, decreased neuronal excitability and reduced neural network activity compared to CTRL hCS. Li rescued excitability of BD hCS neurons by exerting an opposite effect in the two diagnostic groups, increasing excitability in BD hCS and decreasing it in CTRL hCS. We identified 132 Li-associated differentially expressed genes (DEGs), which were overrepresented in sodium ion homeostasis and kidney-related pathways. Moreover, Li regulated secretion of pro-inflammatory cytokines and increased mitochondrial reserve capacity in BD hCS. Through long-term Li treatment of a human 3D brain model, this study partly elucidates the functional and transcriptional mechanisms underlying the clinical effects of Li, such as rescue of neuronal excitability and neuroprotection. Our results also underscore the substantial influence of treatment duration in Li studies. Lastly, this study illustrates the potential of patient iPSC-derived 3D brain models for precision medicine in psychiatry.

## Introduction

Bipolar disorder (BD) is characterized by recurrent episodes of mania and depression and has a prevalence of 1–2% in the general population [1]. The lack of an accurate in vitro model remained a challenge for BD research, but the introduction of induced pluripotent stem cells (iPSCs) [2] has provided a powerful technique for modeling disease etiology and treatment. In particular, patient-derived iPSCs have been demonstrated to be a feasible model for studying in vitro the transcriptional and functional effects of several mood stabilizers commonly prescribed to treat BD [3–5].

Lithium (Li) is considered the treatment of choice for long-term relapse prevention, having a full response rate of ~30% [6], but with a poorly understood mechanism of action. Li has been found to selectively reverse the hyperexcitable phenotype of iPSC-derived neurons from Li-responders (Li-R) [3], and to increase mitochondrial respiration in Li-R patient iPSC-derived neural precursor cells (NPCs) [5]. Additionally, the transcriptional effects of Li have been studied in multiple cell types like peripheral blood mononuclear cells [7], and iPSC-derived neurons [3, 4] and NPCs [5]. Nevertheless, to our knowledge, Li transcriptional effects have not yet been addressed in complex 3-dimensional (3D) cell cultures such as brain organoids derived from BD iPSCs. In recent years, brain organoids have emerged as a promising tool for neurodevelopmental and brain disease modeling in vitro [8–10]. Indeed, 3D cell cultures are regarded as biologically more relevant than 2-dimensional (2D) cell cultures, as they partly recreate in vitro the complex architecture and diverse cell type composition found in vivo [8], allowing for a more informative analysis of the biological processes related to illness and therapeutic interventions.

Since brain cortex abnormalities are strongly associated with BD [11], we specifically generated iPSC-derived human cortical spheroids (hCS) from healthy controls (CTRL) and BD patients, and studied the effects of Li treatment at the transcriptional level by RNA-sequencing analysis and at the functional level by patch-clamp electrophysiology, calcium imaging, mitochondrial assays and cytokine profiling. We hypothesized that Li would differently impact the biochemistry and cellular neurophysiology of CTRL and BD hCS.

## Methods

### Donor samples

Donors were recruited through the Norwegian TOP (Thematically Organized Psychosis) study. Recruitment procedures, inclusion and exclusion criteria and clinical assessments for the TOP study have been described elsewhere [12, 13]. Fibroblasts from skin biopsies were isolated from 10 CTRL and 11 BD patients selected based on clinical information. Patients experiencing no symptoms or only mild symptoms during Li treatment (BD#4–6 and #9–11) were considered Li responders (Li-R), while the other patients (BD#1–3 and #7–8), whose responsiveness to Li was either not confirmed or not known, were considered non-treated (Li-N) (Supplementary Table 1). Li-R group included a partial responder (BD#4), due to periods of poor Li compliance. Details of several donors used in this study (CTRL#1–3 and BD#1–6) have previously been described [5]. Groups were gender-balanced and average age was not significantly different. Supplementary Table 1 summarizes the clinical characteristics of the study participants. All participants have given written consent and the study was approved by the Norwegian Data Protection Agency and the Regional Ethics Committee of the South-Eastern Norway Regional Health Authority (REK grant: #2012/2204). The authors declare that all procedures contributing to this work comply with the ethical standards of relevant guidelines and regulations.

### IPSC derivation and cortical spheroids generation

Fibroblast from all donors were grown, expanded and reprogrammed as previously described [5]. Each iPSC line was subjected to rigorous quality control by phenotyping, regular monitoring of morphology and pluripotency marker expressions. KaryoStat GeneChip array (ThermoFisher) was used for karyotyping of iPSCs at passages 15–20 for digital visualization of chromosome aberrations [5]. All iPSCs were differentiated to cortical spheroids (hCS) following a previously published protocol [8], with proved high reproducibility. Pictures were taken at different time points for determining organoids size progression and Fiji software was used for area counting. Images were converted to 8-bits and binary color, scale was set using as reference distance 51.8 mm of diameter of the culture dish, particles were analyzed counting the area, and volume in mm³ was estimated using the formula: $V = \frac{4}{3}\pi ( {\surd \frac{{{{\rm{area}}}}}{\pi }} )^3$.

### Lithium administration

The hCS from all 21 donors were treated with the clinically recommended therapeutic concentration of 1 mM lithium chloride (Li, Sigma L4408), diluted in sterile Milli-Q water, which was used as vehicle control. Li brain concentration is usually moderately lower than the serum concentration, with an estimated mean brain/serum ratio of 0.78 ± 0.26 [14]. However, we used 1 mM Li for the experiments in accordance with previous in vitro studies [3, 5, 15]. This level is within the therapeutic range (approx. 0.6–1.2 mM) of serum concentrations that are usually targeted during Li treatment of BD patients. Treatment duration was 1 month, from day 150 until day 180, with medium and drug refreshment every 3–4 days. We treated all hCS with Li, regardless of their diagnosis or prescribed treatments, to identify genes and underlying mechanisms associated with Li independent of diagnosis.

### RT-PCR and TaqMan-array cards

At day 180, RNA samples were collected from hCS and cells were lysed with lysis buffer. Total RNA was extracted with the RNeasy Plus Mini kit (Qiagen, #74136). Reverse transcription and RT-PCR and mRNA expression calculation were performed as previously described [5]. Primers used for iPSCs characterization are specified in Supplementary Table 2. For day 180 hCS characterization, it was used a Custom Gene Expression TaqMan Array Card - Format 16 (AppliedBiosystems, #4346798), with primers for detection of the genes in Supplementary Table 3.

### Cryosectioning and immunohistochemistry

CTRL and BD hCS were fixed at day 180 in 4% paraformaldehyde in PBS O/N at 4 °C, transferred to 30% sucrose for 24 h, embedded into optimum cutting temperature (OCT) compound (Sakura Finetek, #4583) and stored at −80 °C. Each line was cut in sections 10 µm thick with a cryostat (Leica) and stainings were performed as previously described [5]. Antibodies and dilutions are specified in Supplementary Table 4. Images were acquired using a Zeiss-LSM700 confocal microscope and processed using Fiji software. At least 300 cells were analyzed for each marker from all lines.

### Electrophysiology

For whole-cell patch-clamp experiments, CTRL and BD hCS were sliced (150 μm thickness) using a vibrating microtome (VT1000S, Leica Biosystems, USA), and slices were kept at room temperature in HEPES-based ACSF composed of 135 mM NaCl, 2 mM KCl, 2 mM CaCl_2_, 1 mM MgSO_4_, 10 mM HEPES, 10 mM d-glucose; pH 7.35; 300–310 mOsm/L. Whole-cell patch-clamp electrophysiology recordings (whole cell currents, potentials and spontaneous excitatory postsynaptic currents, sEPSCs), data evaluation and analysis were performed as previously described [16]. The Nernst equation $V_{{{\rm{rev}}}} = \frac{{RT}}{{zF}}{{{{{\mathrm{ln}}}}}}( {\frac{{[ X ]{{{\rm{out}}}}}}{{[ X ]{{{\rm{in}}}}}}} )$ was used for the theoretical calculation of the reversal potential for Cl^−^ (*V*_rev_) for estimation of potential impact of inhibitory spontaneous synaptic currents to PSCs recording at holding potential −60 mV. The theoretical reversal potential for Cl^−^ in applied experimental conditions was calculated as V rev = −75.61 mV, that does not allow the activation of inward Cl^−^-mediated currents under Vh = −60 mV.

### Live calcium imaging

For live calcium imaging, hCS were sliced similar to patch-clamp with a vibrating microtome (150 μm thickness). Slices were allowed to recover for at least 1 h at room temperature in HEPES-based ACSF composed of 135 mM NaCl, 2 mM KCl, 2 mM CaCl_2_, 1 mM MgSO_4_, 10 mM HEPES, 10 mM d-glucose pH 7.35 with an osmolarity of 310 mOsm. For Ca^2+^ imaging, slices were loaded with 1 μM Cal-520, AM (Abcam). Recordings, data evaluation and analysis were performed as previously described [17]. A custom-made MATLAB (Math Works, USA) code was used to analyze calcium traces (deposited in Github: https://github.com/Zluglu/CalciumSignalAnalysis.git). Two hundred and ninety-one recordings of calcium transients (102 CTRL and 189 BD) were generated and 50–200 ROIs per recording were analyzed.

### RNA extraction, sequencing and data processing

Total RNA was extracted from hCS using the RNeasy Plus Mini Kit (Qiagen). Library preparation and paired-end RNA-sequencing were performed at the Norwegian High-Throughput Sequencing Centre (www.sequencing.uio.no). Briefly, libraries were prepared with Illumina TruSeq Stranded mRNA kit, and sequenced as a single batch on an Illumina NovaSeq S4 platform at an average depth of 50 million reads per sample, using a read length of 100 bp and an insert size of 350 bp. Raw sequencing reads were quality assessed with FastQC (Babraham Institute) and further processed with Trimmomatic V0.32 [18]. HISAT2 [19] was then used to map the trimmed reads to the human GRCh38 reference genome. To quantify gene expression levels, mapped reads were summarized at the gene level using featureCounts [20] guided by ENSEMBL annotations.

### Cell type deconvolution and comparison with in vivo spatiotemporal brain expression

Computational estimation of cell type abundances (deconvolution) was performed with CIBERSORTx [21] using the web-interface (https://cibersortx.stanford.edu/) with default parameters, selecting 500 permutations for statistical testing. We used as reference the expression signature from ten cell types obtained from single-cell RNA sequencing of three of our 180 days old CTRL hCS using the Chromium Next GEM Single Cell 3′ v3.1 (10X Genomics). Data from the CTRLs was integrated into a merged dataset, consisting of 11,649 cells. Normalization and integration were performed with Harmony and Seurat v4 SCT. Cluster annotation was performed with GSEA together with several automatic cell type prediction packages. To assess the extent to which in vitro hCS transcriptional profiles match the gene expression profiles of human primary in vivo brain tissue, gene-level spatiotemporal RNA sequencing data sets were downloaded from the BrainSpan resource (https://www.brainspan.org/static/download.html) [22, 23]. Concordance analyses were performed using Spearman’s correlation after converting gene counts to RPKM values and filtering out non-expressed and low-abundance transcripts.

### Differential expression (DE) analysis and gene ontology (GO) enrichment tests

Genes were pre-filtered using filtByExpr function in edgeR package [24], keeping genes with a count per million above *k* in *n* samples, where *k* is determined by the library size and *n* is determined by the smallest group sample size [25]. Since KaryoStat analysis revealed chromosomal aberrations in several of the iPSC lines (Supplementary Fig. 1), genes located within affected regions were excluded prior to analysis (642 genes; Supplementary Table 5). For DE analyses, the R packages DESeq2 [26] and limma-voom [27] were used. While DESeq2 typically identifies a larger number of differentially expressed genes (DEGs) than limma-voom, the latter allows for more complex design matrices. To increase the number of significant genes, any DEG identified by either of the two packages was considered significant and retained for downstream analyses. This strategy was deemed appropriate as the two methods were found to yield highly concordant results (Supplementary Fig. 6a, b). Since a paired design was used in all DE analyses comparing Li-treated hCS against non-treated hCS, demographic variables were perfectly matched across groups and only neuronal proportions were adjusted for. Otherwise, sex and age were included as covariates in addition to neuron abundance. A DEG was considered significant if the FDR was <0.10. Gene Ontology (GO) enrichment tests of significant DEG sets were conducted with clusterProfiler [28] selecting BP (biological processes) as the ontology of interest. A GO term was considered significantly enriched if the FDR was <0.10. We performed gene set enrichment analysis (GSEA) using the R package clusterProfiler [28] and enrichment analysis of synaptic functions using SynGO (https://www.syngoportal.org/).

### ELISA cytokine quantification

Supernatants were collected at different time points from all 21 donor hCS (10 CTRL and 11 BD) and stored at −80 °C until analysis. Samples were not diluted before analysis. Cytokine levels in supernatants were measured with U-PLEX Biomarker Group 1 (hu) Assay (MSD, K15067L-1), customized for detection of human IL-1β, IL-6 and TNF-α. Cytokine concentration values were corrected by the estimated volume (in cm^3^) of hCS tissue in each plate estimated with ImageJ as indicated in the section for hCS.

### Bioenergetic assessment

Cellular bioenergetics assessment of hCS was performed using a Seahorse XFe24 extracellular flux analyzer (Agilent SeahorseXF Analysers) [5]. Briefly, hCS were sliced (150 μm thickness), and allowed to recover for 5–10 minutes at room temperature in HEPES-based ACSF. Slices were transferred to unbuffered media in a XFe24-well plate coated with poly-L-lysine (Sigma, #P8920), and 5–6 pieces were plated per well. Mitochondrial respiration function was recorded as previously described [5]. All experiments were performed in technical duplicates and repeated at least twice. Values were normalized to the total protein content in each well of the plate using the BCA protein assay kit (ThermoFisher, #23225).

### Statistical analysis

One batch of organoids was differentiated for each of the 21 donor hiPSCs, and two organoids per donor were used for each read-out, except for RNA-seq analysis, which was performed using one organoid per donor and condition. Data are presented as mean ± SD for organoid size and IHC, and as mean ± SEM for electrophysiology, calcium imaging, Seahorse and cytokine analysis. Statistical significance was tested using either one-way analysis of variance (ANOVA) between groups for the transcriptional analysis or ANCOVA for spatiotemporal comparisons. Mann-Whitney U test and Wilcoxon test were used for unpaired and paired group comparisons respectively for calcium imaging, whole-cell patch clamp electrophysiology, mitochondrial parameters, hCS size and IHC quantifications. As the distribution of cytokine levels was skewed, data was log10 transformed before performing the univariate regression analysis for assessing cytokine levels at different time points evaluating time, group, and time × group effects. Regression model between cytokine and mitochondrial values with estimated cellular fractions and hCS size was performed by first carrying out a partial regression adjusting for donor id, treatment and diagnosis group (i.e. patient or control) as covariates. Variables with *p* < 0.2 were then included in a stepwise regression, where donor id, treatment and diagnostic group were also included and adjusted for.

## Results

### Differentiation of CTRL and BD iPSCs to hCS

**Fig. 1:**
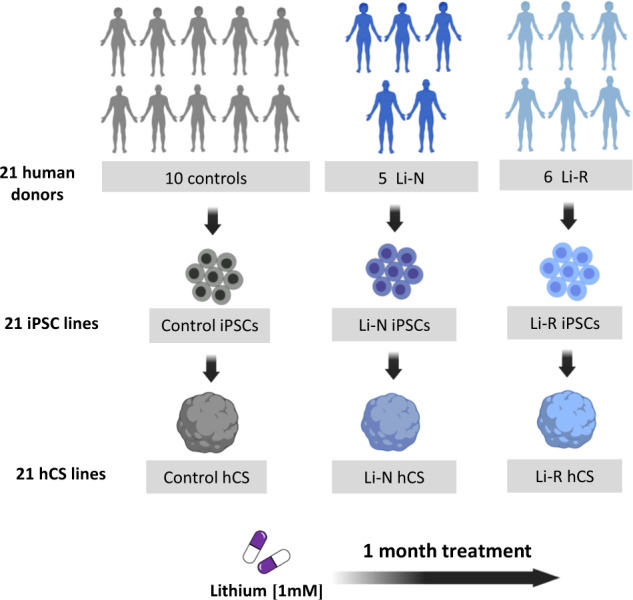
Schematic representation of the experimental design for Li treatment of hCS. Fibroblasts isolated from 10 controls (CTRL) and 11 bipolar disorder (BD) patients, including 5 lithium non-treated (Li-N) and 6 Li-responders (Li-R), were reprogrammed to induced pluripotent stem cells (iPSCs) and differentiated to cortical spheroids (hCS). All 21 hCS lines were treated with 1 mM lithium (Li) and water as vehicle control. Medium-to-long-term effects of drug exposure (1 month) were assessed through transcriptional profiling and functional analyses in 180-day-old hCS.

**Fig. 2:**
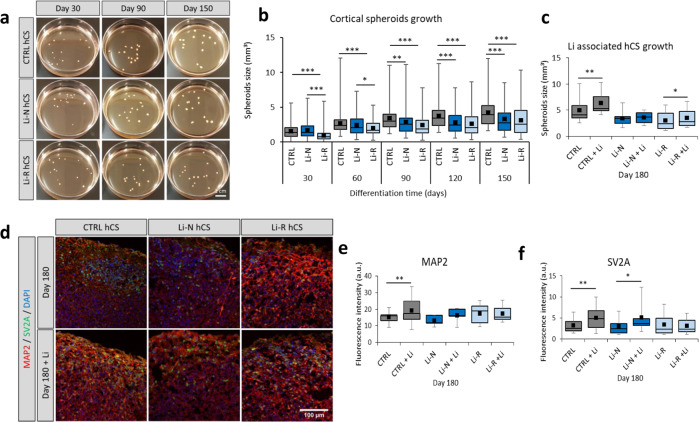
Characterization of Li treated hCS. **a** Representative images of CTRL and BD hCS from both Li-N and Li-R patients at days 30, 90 and 150 of differentiation. **b** Growth of CTRL and BD hCS size through differentiation show decreased volume at most time points of Li-N and Li-R hCS compared to CTRL hCS. Individual hCS volumes were estimated from their areas measured with ImageJ. **c** Li induced growth after 1 month treatment from day 150 until day 180. Both CTRL and Li-R, but not for Li-N hCS, present a significant increase in size. **d** Representative immunohistochemistry images of CTRL, Li-N and Li-R hCS with and without Li treatment. Fluorescence was measured for cytoplasmic MAP2 (**e**) and SV2A (**f**), by counting signal intensity with ImageJ. Results are presented in arbitrary units (a.u.). CTRL (*N* = 10), Li-N (*N* = 5), Li-R (*N* = 6). Data was analyzed by Mann–Whitney *U* test and Wilcoxon test for unpaired and paired group comparisons respectively. *p < 0.05, **p < 0.01, ***p < 0.001.

Fibroblasts from skin biopsies were obtained from 10 CTRL and 11 BD patients, including 5 Li-N and 6 Li-R donors, and reprogrammed to iPSCs. One clone from each donor was differentiated to hCS (Figs. 1 and 2a). Details about donor age, gender and diagnosis are provided in Supplementary Table 1.

All 21 iPSC lines expressed pluripotency markers OCT4, SOX2, NANOG and alkaline phosphatase (AP) (Supplementary Fig. 1a–g). Karyotyping revealed a partial loss in three patient lines at the 6q24.3 locus (Supplementary Fig. 1f and Supplementary Table 5). Furthermore, another patient line harbored a 98.7 kb and a 39.6 kb gain in the 1q21.3 and 5p15.31 loci respectively, and a CTRL line a 6.97 kb loss in the 6p24.3 locus (Supplementary Fig. 1c). These chromosome gains and losses included ~642 genes with detectable expression levels in the hCS (Supplementary Table 5). Consequently, these genes were excluded before conducting the DE analyses.

Average size of the BD hCS was lower than CTRL hCS, in particular at later stages of differentiation (Fig. 2b), in line with MRI findings of thinner cortical regions in BD [11, 29]. At day 180 of differentiation, RT-PCR analysis of cortex development and maturation markers revealed decreased expression in *CTIP2*, *SATB2*, *TBR1* and *SYN1* in Li-N hCS compared to CTRL hCS (Supplementary Fig. 2a).

All hCS were treated with 1 mM Li for 1 month from differentiation day 150–180. This treatment duration was chosen because full therapeutic effects typically require at least 10–21 days [30]. Li treatment produced a significant increase in size in both CTRL and Li-R hCS (Fig. 2c), in line with studies showing that Li increases neurogenesis in the dentate gyrus in rodent hippocampus [31], and is associated with thicker cortex and larger subcortical volumes in BD patients [32].

Immunohistochemistry of day 180 hCS revealed no differences in the synaptic density marker synaptic vesicle protein 2A (SV2A) nor the neuron maturity marker microtubule-associated protein 2 (MAP2) between BD and CTRL (Fig. 2d–f), but Li increased MAP2 and SV2A levels in CTRL hCS and increased SV2A in Li-N hCS (Fig. 2e, f), suggesting improved neuronal maturation.

### Li treatment rescues neuronal excitability in BD hCS

**Fig. 3:**
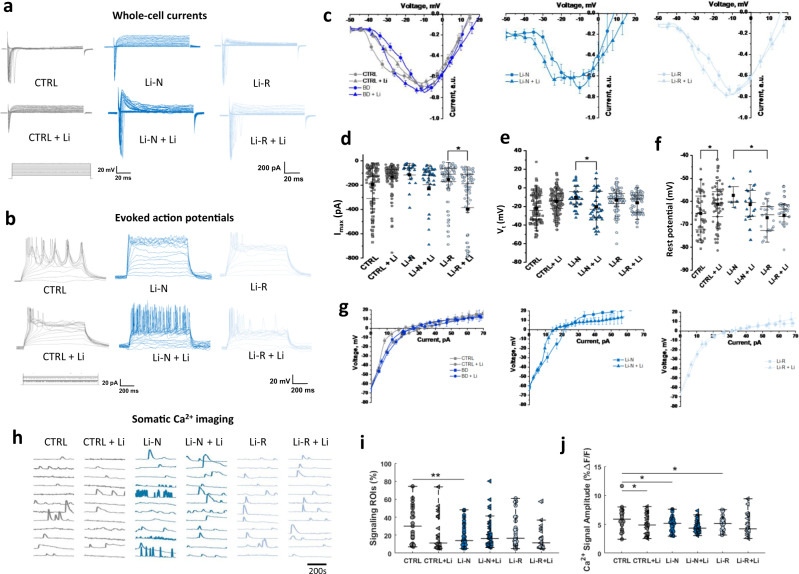
Patch-clamp electrophysiology and calcium imaging of hCS neurons. **a** Raw traces of whole-cell currents (holding potential Vh = −60 to −70 mV) representative for each condition. **b** Raw traces of action potentials (AP) representative for each condition. **c** Current–voltage (*IV*) curves for each group. Currents of individual IV curves were normalized to maximum peak values and then averaged. **d** Evoked whole-cell currents peak maximum values (*I*_max_). Li significantly increases *I*_max_ in BD hCS (Δ(*I*_max_) = 171.94 ± 76.80 pA, *p* = 0.01), particularly for Li-R hCS neurons (Δ(*I*_max_) = 209.34 ± 102.42 pA, *p* = 0.03). **e** Threshold membrane voltages (*V*_t_) of *I*_max_, showing BD is less excitable than CTRL hCS neurons (Δ(*V*_t_) = 5.32 ± 2.93 mV, *p* = 0.04). Li treatment increases excitability of BD hCS (via decreasing threshold voltage, Δ(*V*_t_) = 5.14 ± 2.86 mV, *p* = 0.02), significant for Li-N hCS (Δ(*V*_t_) = 11.84 ± 5.62 mV, *p* = 0.02). **f** Membrane resting potentials (RP). **g** Voltage–current (*VI*) curves for each group. Individual *VI* curves were averaged for correspondent group. **h** Representative trace images of spontaneous CaCl_2_ transients in hCS regions of interest (ROIs) over a 20-min period. **i** Percentage of signaling ROIs after Li treatment. **j** Average amplitude of Ca^2+^ signal transients (%Δ*F*/*F*). I-shaped box charts represents 25–75% of range intervals and median horizontal brackets, overlapped with data; square symbol represent mean value. CTRL (*N* = 10), Li-N (*N* = 5), Li-R (*N* = 6). Data was analyzed by Mann–Whitney *U* test and Wilcoxon test for unpaired and paired group comparisons respectively. *P < 0.05, **P < 0.01.

We performed whole-cell patch-clamp recordings of 180-day-old hCS to characterize hCS neurons and functionally assess diagnosis-specific differences and Li treatment effects in vitro (Fig. 3a–g and Supplementary Fig. 2b–h). Evoked whole-cell currents (Fig. 3a) demonstrated that BD hCS neurons were less excitable than CTRL hCS neurons, with right-shifted current–voltage (*IV*) curves (Fig. 3c) toward higher membrane depolarization values (threshold voltage, *V*_t_, Fig. 3e and Supplementary Table 6) required for maximum whole-cell current (*I*_max_) induction. Li-N and Li-R showed similar characteristics in IV curves. Li treatment had an opposite effect in CTRL and BD hCS neurons, decreasing excitability in CTRL hCS (right-shifting *IV* curves and *V*_t_ shift, not significant) and increasing excitability in BD hCS (left-shifting *IV* curves, Fig. 3c and Supplementary Fig. 2b) and decreasing *V*_t_ significantly in Li-N hCS (Fig. 3e, Supplementary Fig. 2e). Li treatment increased currents in BD hCS, mainly driven by effects in Li-R hCS (Fig. 3d, Supplementary Fig. 2d and Supplementary Table 6).

In line with voltage-clamp recordings, current-clamp recordings (Fig. 3b) revealed similar excitability differences (CTRL hCS are more excitable due to left-shifted voltage-current (VI) curves, Fig. 3g) and changes upon treatment (Li decreased excitability in CTRL hCS and increased it in BD hCS). Evoked action potentials (AP) appeared at similar amplitudes of threshold currents (I_t_), and Li treatment did not affect *I*_t_ (Supplementary Fig. 2h). Despite comparable membrane resting potentials (RP) for CTRL and BD hCS (Fig. 3f and Supplementary Fig. 2f), Li significantly decreased RP only in CTRL hCS. Input resistance (*R*_in_) was analogous between groups, and Li treatment had no significant effect.

Next, we studied Li effects on synaptic activity and connectivity by recording spontaneous excitatory postsynaptic currents (sEPSC), and performed distribution analysis for sEPSC amplitudes and frequencies (Supplementary Fig. 3a–k and Supplementary Table 7). Li-R exhibited higher sEPSC amplitudes compared to CTRL and Li-N, and sEPSC frequencies were similar between groups. Li treatment resulted in a non-significant increase of sEPSC amplitudes for CTRL and decrease for Li-R hCS, and non-significant increase of sEPSC frequencies in all groups, indicating a tendency of increased synaptic activity.

To assess whether the decreased excitability of single neurons in BD hCS (Fig. 3c, g) could modify neural network activity we measured somatic Ca^2+^ transient events (Fig. 3h–j and Supplementary Fig. 4a–j). In untreated hCS, BD hCS showed significant decrease in percentage of signaling cells per region of interest (ROI), driven by the Li-N group (Fig. 3i and Supplementary Fig. 4a) and the amplitude of signal events compared to CTRL hCS (Fig. 3j and Supplementary Fig. 4b), suggesting decreased neural network activity. Li treatment decreased Ca^2+^ signal amplitudes in all groups, but this was only significant in CTRL hCS (Fig. 3j).

### Cell type deconvolution and spatiotemporal gene expression analysis of hCS

**Fig. 4:**
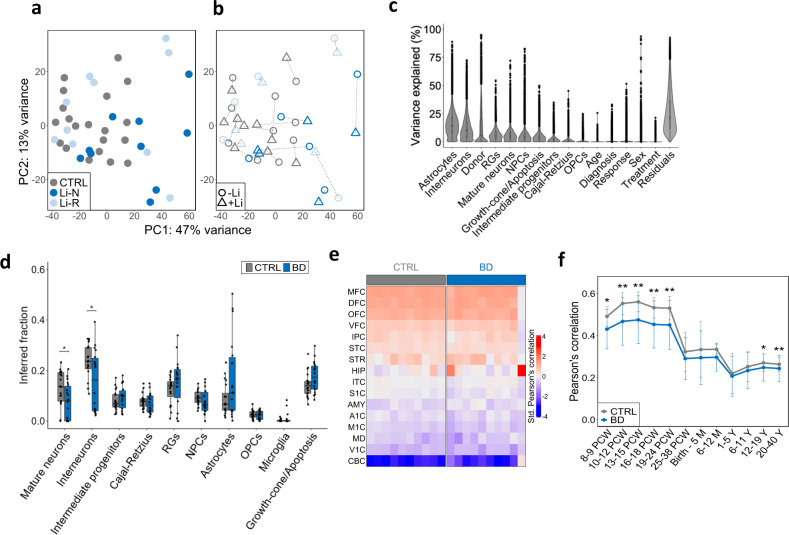
Transcriptomic characterization and spatiotemporal gene expression analysis of hCS. **a** Principal component analysis (PCA) showing a strong donor effects and weaker clustering across diagnostic and response categories. **b** The same PCA plot displaying pairing information between untreated (circles) and treated (triangles) pairs. **c** Variance partition plot showing the proportion of gene expression variance attributed to different sources. Residuals constitute additional, unknown sources of variation not accounted for. **d** Estimation of cell type fractions in untreated CTRL and BD hCS. Significant differences detected in mature neurons and interneurons proportions. Data was analyzed by Mann–Whitney *U* test for group comparisons. **e** Overlap between in vitro gene expression (hCS) and in vivo human spatial brain expression profiles (BrainSpan). Brain regions nomenclature and abbreviations is the same as in [71]. **f** Overlap between in vitro gene expression and in vivo human temporal brain expression profiles. Strong correspondence seen between hCS and mid fetal stages (*p < 0.05, **p < 0.01, ANCOVA). CTRL (*N* = 10), Li-N (*N* = 5), Li-R (*N* = 6). OPCs oligodendrocyte precursor cells, RGs radial glia cells, NPCs neural precursor cells.

We next assessed the effect of Li on global gene expression. Principal component analysis (PCA) showed weak clustering of samples across diagnostic and response groups (Fig. 4a). Moreover, evaluation of treated and untreated pairs showed that Li treatment had mostly small effects across donors (Fig. 4b). This was confirmed by variance partition analysis, which attributed only small effects to Li responsiveness and gender (Fig. 4c). As expected, cell type composition and donor effects were the most important sources of variation in gene expression (Fig. 4c).

Computational deconvolution of cell type proportions showed that CTRL hCS had a substantial increase in mature neurons and interneurons compared to BD hCS (Fig. 4d). Response-status showed significant effect on several cell fractions in Li-N hCS, including reduced neuronal types and increased RGs and intermediate progenitors, while Li treatment did not have a significant effect on cell type estimates (Supplementary Fig. 5a, b).

To determine the extent to which hCS gene expression profiles resemble human developmental brain profiles in vivo, spatiotemporal comparisons were performed using the BrainSpan dataset [22, 23]. Both CTRL and BD hCS showed strongest overlap with the medial, dorsolateral and orbitofrontal prefrontal cortices (MFC, DFC and OFC) (Fig. 4e). Furthermore, all hCS showed transcriptional similarities with early fetal brain tissue of 8–24 post-conception weeks (Fig. 4f). Treatment and response-status had no significant effect on the spatiotemporal overlap with in vivo brain profiles (Supplementary Fig. 5c–f).

### Li-associated genes are involved in sodium ion homeostasis and kidney-related functions

**Fig. 5:**
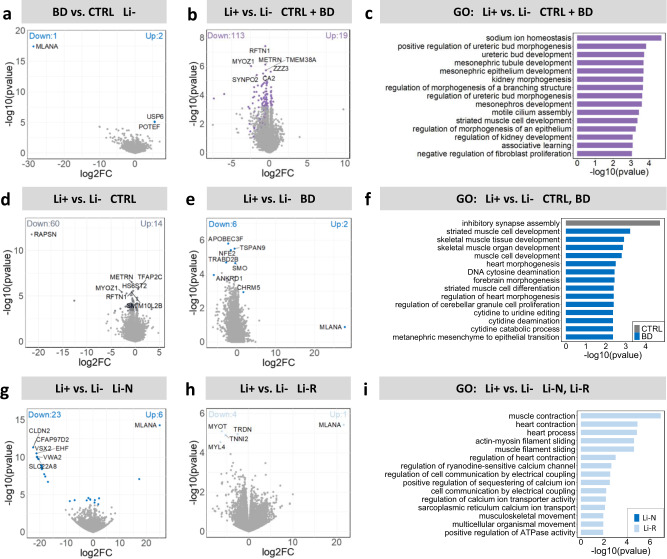
Li-associated DEGs and GO enrichment analysis of hCS stratified by diagnostic and response status. **a** Volcano plot of all DEGs identified for the untreated (Li-) BD vs. CTRL hCS. **b** Volcano plot of all DEGs identified for the treated (Li+) vs. untreated (Li−) hCS, independent of diagnosis. **c** Gene ontology (GO) pathway analysis (biological processes) of the 132 Li-associated DEGs identified in all samples independent of diagnosis. **d**, **e** Volcano plots of all Li-associated DEGs identified in CTRL and BD hCS, respectively. **f** GO pathway analysis of the Li-associated DEGs (74) that were unique to CTRL hCS (gray) and DEGs (8) that were unique to BD hCS (blue). **g**, **h** Volcano plots of all Li-associated DEGs in Li-N and Li-R hCS, respectively. **i** GO pathway analysis of the Li-associated DEGs [29] that were unique to Li-N hCS (dark blue) and DEGs [5] that were unique to Li-R hCS (light blue). CTRL (*N* = 10), Li-N (*N* = 5), Li-R (*N* = 6).

To investigate how Li modulates gene expression, and whether this modulation differs across disease and Li response status groups, six sets of differential expression (DE) analyses were performed as described in the methods. (i) Comparing untreated BD and CTRL hCS identified only 3 DEGs, and of these *MLANA* was downregulated while *USP6* and *POTEF* were upregulated in BD hCS (FDR < 0.1) (Fig. 5a) (SupplementaryTable 8). (ii) Comparing untreated and Li treated hCS from all 21 donors identified a relatively small number of 132 significant Li-associated DEGs (FDR < 0.1) (Fig. 5b, Supplementary Tables 9, 10 and Supplementary Fig. 6a, b). GO pathway enrichment analysis showed that these genes were enriched for the *sodium ion homeostasis* pathway and several kidney-related pathways (*p* < 0.05, FDR < 0.1) (Fig. 5c and Supplementary Table 11). (iii) Comparing untreated and Li treated CTRL hCS identified 74 Li-associated DEGs (Fig. 5d and Supplementary Table 12). The genes were exclusively enriched for the GO term *inhibitory synapse assembly* (adjust *p* < 0.05, FDR < 0.1) (Fig. 5f), and the enrichment was mainly driven by upregulation of *LGI2*, *GABRA1* and *CBLN4*. SynGo analysis showed that these genes were enriched for the *synaptic vesicle* cellular component (Supplementary Fig. 6c). (iv) Comparing untreated and Li treated BD hCS identified 8 Li-associated DEGs (Fig. 5e). Li upregulated *MLANA* and *CHRM5* and downregulated *APOBEC3F*, *TSPAN9*, *NFE2*, *TRABD2B*, *SMO* and *ANKRD1* (Fig. 5e). These genes are mainly involved in muscle cell development processes (Fig. 5f), with the main drivers being *SMO* and *ANKRD1*. (v) Comparing untreated and treated Li-N hCS identified 29 Li-associated DEGs (FDR < 0.1) (Fig. 5g and Supplementary Table 13). None of the genes were significantly enriched for any GO term. *vi)* Finally, comparing untreated and treated Li-R hCS identified 5 Li-associated DEGs (FDR < 0.1) (Fig. 5h). Again, *MLANA* was upregulated, while *MYOT*, *TRDN*, *TNNI2* and *MYL4* were downregulated after Li treatment (Fig. 5h). These genes were significantly enriched for GO terms mostly related to excitable tissue functions like *muscle contraction* or *muscle filament sliding* (Fig. 5i). We additionally performed GSEA to further elucidate the affected biological processes, and the results were similar to the GO analysis findings (Supplementary Fig. 6d–f). Notably, we identified three overlapping genes between DEGs identified in this study and BD-associated risk genes [33], i.e. *CNTN5*, *DOCK2* and *MAD1L1*, which are involved in the formation of axonal connections, actin skeleton remodeling, and cell cycle control, respectively.

### Li treatment increases IL-1β secretion in CTRL and BD hCS and decreases TNF-α in CTRL hCS

Systemic levels of the pro-inflammatory cytokines IL-1β, IL-6 and TNF-α are elevated in BD [34–36], and some studies suggest Li may have a modulatory effect [37, 38]. Hence, we hypothesized that these cytokines could be increased in supernatants from BD hCS compared to CTRL during differentiation, and that Li treatment would further regulate their levels.

**Fig. 6:**
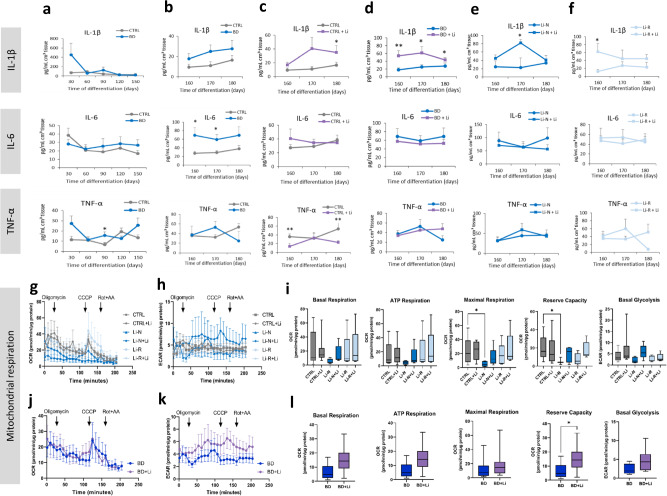
Cytokine analysis and Seahorse bioenergetics of Li treated hCS. **a**, **b** IL-1β, IL-6 and TNF-α cytokine levels in hCS at different time points. Li treatment effect in CTRL hCS (**c**), in BD hCS (**d**), and stratified in Li-N (**e**) and Li-R (**f**). Data is presented as mean ± SEM. **g** Seahorse oxygen consumption rate (OCR) kinetics graph. **h** Extracellular acidification rate (ECAR) kinetics graph showing glycolysis activity. **i** OCR parameters and ECAR basal glycolysis after Li treatment for each condition. **j** OCR and **k** ECAR kinetic graphs for Li treatment of BD hCS. **l** OCR parameters and basal glycolysis after Li treatment in BD hCS. All Seahorse experiments were run in quadruplicates, and values were normalized to total protein. Mitochondrial parameters were normalized to total protein content and data is presented as mean ± SEM. CTRL (*N* = 10), Li-N (*N* = 5), Li-R (*N* = 6). Mann–Whitney *U* test and Wilcoxon test were used for unpaired and paired group comparisons respectively. *p < 0.05, **p < 0.01.

Cytokine profiling of supernatants collected at different time points from day 30 to day 180 showed no difference between BD and CTRL hCS for IL-1β (Fig. 6a, b), but BD hCS had increased TNF-α at day 90 (Fig. 6a) and increased IL-6 at days 160 and 170 compared to CTRL hCS (Fig. 6b). Regression analysis for BD hCS showed a group effect (*p* = 0.028), and group-by-time interaction (*p* = 0.041) for TNF-α and group-by-time interaction effect for IL-1β (*p* = 0.026), highlighting the relevance of these two pro-inflammatory cytokines in BD etiology. Stratifying results based on patient response-status showed decreased IL-6 levels in day 30 Li-N hCS compared to CTRL hCS (Supplementary Fig. 7a), and this trend was reversed after day 60 (Supplementary Fig. 7a). Response status had no consistent effect on basal cytokine levels in the untreated groups at days 160, 170 and 180 (Supplementary Fig. 7b).

In CTRL hCS, Li treatment increased IL-1β at day 180, and decreased TNF-α at days 160 and 180 (Fig. 6c). On the other hand, in BD hCS, Li increased IL-1β at days 160, 170 and 180, (Fig. 6d). Li increase of IL-1β in BD hCS seems to be independent of Li-responsiveness, as both Li-N and Li-R showed increased IL-1β at different time points (Fig. 6e, f). No significant difference in IL-6 secretion was detected for any of the treated groups. Interestingly, regression analyses revealed a positive correlation of proinflammatory cytokines with astrocytes and microglia cell fractions, and a negative correlation with neuronal cell types, RGs and OPCs fractions (Supplementary Fig. 7c).

### Li increases mitochondrial reserve capacity in BD hCS

Next, we performed mitochondrial characterization of oxygen consumption rate (OCR) and extracellular acidification rate (ECAR) (Fig. 6g–l). Untreated Li-N hCS presented decreased maximal respiration capacity compared to CTRL hCS, and Li treatment rescued the normal values in Li-N hCS (Fig. 6i). Moreover, Li treatment for 1 month increased reserve capacity in BD hCS (Fig. 6l), regardless of Li-responsiveness. Interestingly, Li treatment did not alter these parameter in CTRL hCS (Fig. 6i). All analyzed bioenergetics parameters displayed an increasing trend after Li treatment (Fig. 6g–l), and basal glycolysis was significantly increased after treatment when combining all CTRL and BD donors (Supplementary Fig. 7d–f).

Lastly, we previously showed decreased gene intron retention rate (IRR) after 6 h treatment with Li in NPCs, which disappeared after 1 week of treatment [5]. Here, we calculated IRR values for all hCS, and found no significant reduction after 1 month treatment neither in CTRL nor BD hCS (Supplementary Fig. 7g, h), consistent with our finding that Li-induced reduction in IRR occurs during the first hours of treatment and rapidly fades away.

## Discussion

Genome-wide association studies (GWAS) have identified 64 risk loci for BD [33]. Risk alleles are enriched in genes involved in synaptic signaling pathways, with high specificity of expression in neurons of the prefrontal cortex and hippocampus [33]. However, there is still limited understanding of which specific biological features are associated with diagnosis, response status and Li treatment. IPSC models used in our experiments may bridge the gap between genotypes and disease phenotypes, as they preserve the genetic signature of patient donors and allow in vitro experimentation with a myriad of conditions and drug treatments.

### Treatment impact—effect of duration

A pioneer study showed that 1 week Li treatment regulated 560 genes in human iPSC-derived neurons from Li-R BD patients [3]. Here, after 1 month Li treatment in hCS, we identified a relatively small number of 132 Li-associated DEGs. In our previous studies, Li exposure for 4 days in rats significantly regulated 1108 genes [39], while 6 h treatment in human NPCs regulated 9557 genes, none of which remained significant after 1 week of treatment [5]. Further studies of Li effects in peripheral blood from BD patients found only 56 genes that were regulated after 2–8 weeks treatment [40]. Similarly, reduction in IRR seen after 6 h treatment duration with Li disappeared after 1 week in NPCs [5] and 1 month treatment in our hCS. These results seem to indicate that Li has a substantial short-term impact on gene expression, which in the middle-to-long-term is reversed and homeostasis is restored, likely by compensatory mechanisms or negative-feedback loops, illustrating the heavy influence of treatment duration in Li studies. This duration effect might also explain lack of identification of classical Li-associated genes, such as *BCL2*, *AP1* or *GSK3B*. Although discrepancy of results with previous studies might be the result not only of biological, but also technical factors [41].

### Treatment impact—neuroprotection

Accumulating evidence points to a neuroprotective role of Li in the brain [42–44]. Li increases hippocampal and cortical size [31, 32], slows cognitive deterioration [42], preserves cognition in responder patients [43], and its deficiency causes behavioral abnormalities in rats [45]. We found that Li increased CTRL and Li-R hCS size, and increased expression of MAP2 in CTRL hCS and SV2A in both CTRL and Li-N hCS, consistent with findings showing that Li increases MAP2 expression in neural stem cells [46], as well as neurogenesis in animal models [31]. Although most in vitro studies are conducted with cells from patients on Li medication, our results in CTRL hCS suggest that Li neuroprotective effects are independent of diagnosis, in line with findings that long-term low-dose dietary Li intake is associated with longevity in the general population [47, 48].

Electrophysiology and calcium signaling characterization revealed that BD hCS exhibited decreased neuronal excitability and lower neural network activity compared to CTRL hCS. Li treatment increased excitability and rescued decreased currents in BD hCS and had an opposite effect in CTRL hCS. This differs from the findings in previous publications, where increased neuronal excitability and hyperactive neural network activity was found in hippocampal dentate gyrus-like iPSC-derived neurons from BD patients [3, 4]. However, hyperexcitability was only seen in 3-week-old neurons, and Mertens et al. also reported that more mature 8-week-old BD neurons reversed the abnormal excitability to decreased neuron excitability and lower Na^+^ current amplitudes [3], which is in line with our results. Notably, enhanced accumulation of Li was reported in neurons derived from BD patients compared with CTRL [49], and in dendritic spines Li displaces intracellular Na^+^ concentrations, thereby reducing its concentration [50]. This accumulation might explain why Li prophylactic action is not immediately reversed upon discontinuation of treatment in BD patients [51]. Moreover, since Li enters neurons primarily through the voltage-gated Na^+^ channels, it is more likely to affect hyperactive neurons [50].

### Treatment impact—side effects

A narrow therapeutic index [52] and several adverse effects restrict Li benefits, and particularly kidney dysfunction is an important contra-indication for long-term Li-prescription [53, 54]. In our DE analysis, we identified several Li-associated DEGs involved in kidney-related processes. Although the enrichment of these pathways was driven by a relatively small number of genes, all of them are expressed at low-medium levels in the brain (https://www.gtexportal.org/). Given that our hCS model contains mainly neurons and glial cells, these findings indicate that regulation of kidney-related genes by Li is systemic and not restricted to the kidneys. While these results concord well with the known side effect burden of Li, their physiological significance in the brain remains unclear.

We also found that Li-associated DEGs are enriched in *ion homeostasis* pathway. This is consistent with the fact that BD patients under Li treatment are required to monitor sodium chloride (NaCl) intake because any sudden variation of ingested Na^+^ can affect Li levels. NaCl supplementation diminishes Li side effects [55], and may stabilize serum Li concentration [56], while Li treatment can normalize elevated intracellular Na^+^ [57].

### Etiology insights—ankyrins and Na^+^ channels dysregulation

Two DEGs associated with diagnosis and Li-treatment in BD hCS, *POTEF* and *ANKRD1* respectively, encode proteins containing ankyrin domains. Variation in genes encoding proteins with ankyrin repeats (*TRANK1*, *SHANK2* and *ANK3*), is one of the strongest genetic findings in BD [33, 58–60]. Ankyrins regulate assembly and anchoring of numerous proteins, including voltage-gated Na^+^ and K^+^ ion channels [61–63]. Healthy neurons have high densities of voltage-gated ion channels clustered in the axon initial segments (AIS) and nodes of Ranvier [62, 64, 65], suggesting that defects in genes coding for ankyrin proteins might alter neuronal activity in patients. Decreased Na^+^ currents could be indicative of decreased Na^+^ channels embedding and clustering, which could affect excitability in BD hCS, including right-shifted *IV* curves and altered thresholds for currents and APs.

The canonical ankyrins ANK1-3 contain 23-24 ankyrin repeats, but a mapping study of ANK3 fragments demonstrated that repeats 1 to 6 are sufficient for binding Na^+^ channels [63]. Mutational analysis of these repeats showed a critical role of two lysine residues to bind Na_v_1.2 [63], which is encoded by another BD-associated gene, *SCN2A* [33]. Remarkably, POTEF contains 7 ankyrin repeats and has the same critical lysine residues in the same β-hairpin tip between repeats 2-3. Thus, we suggest that upregulated *POTEF* in untreated BD hCS could reflect a compensatory mechanism to replace defective ankyrins in patients.

### Etiology insights—role of pro-inflammatory cytokines

BD pathogenesis is associated with dysregulation of the pro-inflammatory cytokines TNF-α, IL-6 and IL-1β [34–36, 66], of which TNF-α and IL-6 are increased during manic and depressive episodes [34, 66]. Here, univariate regression analysis showed a significant increase in TNF-α in BD hCS for all time points. TNF-α regulates apoptotic cascades [67], which may be related to neuronal loss, potentially explaining the reduction in neurons in BD hCS compared to CTRL hCS. Moreover, Li treatment increased IL-1β secretion in both CTRL and BD hCS, in line with most previous studies in immune cells [37, 38, 68]. IL-1β is known to have neurotrophic effects in the mammalian brain, such as neuroprotection [69], enhanced neurogenesis [70] and improved migration of cortical neurons [71], suggesting that the neuroprotective effects of Li may, at least partly, be mediated by IL-1β.

### Etiology insights—mitochondrial dysregulation

We previously reported that, in Li-R NPCs, Li treatment increased mitochondrial maximal respiration and reserve capacity [5]. Here, we confirmed that Li significantly increased reserve capacity in BD hCS. However, we also found a deficit of maximal respiration in the Li-N group specifically, and a general effect of Li on mitochondrial function of BD hCS independent of Li responsiveness status. Possible explanations for this discrepancy between our NPCs and hCS findings include the extension of treatment duration from 1 week to 1 month, the greater cell-type complexity of 3D hCS relative to 2D NPCs, and the larger number of donors in the present study.

## Limitations

This study has three main limitations. First, the sub-optimal clinical phenotype of patient’s responsiveness to Li, as we were not able to retrospectively confirm lack of Li response in all Li-N donors due to insufficient information in the clinical records. Second, while the hCS model was able to recapitulate key features of human corticogenesis, hCS architecture is rather rudimentary and have a modest electrophysiological activity in comparison to the adult brain cortex. Third, the average age-of-onset of BD is about 25, while gene expression spatiotemporal comparison showed that hCS resemble fetal cortical tissue of 8–24 weeks post-conception, adding a note of caution when interpreting the results. Thus, the disease-related molecular effects captured in the hCS likely stem from predisposing biological factors.

## Conclusions

We found that BD hCS had decreased neuronal excitability at the single-cell and neural network levels compared to CTRL hCS, and Li treatment rescued neuronal excitability in BD hCS. Transcriptional profiling revealed that Li treatment regulated genes enriched for biological processes related to sodium ion homeostasis and renal functions. Additionally, Li increased IL-1β protein secretion in CTRL and BD hCS and decreased TNF-α secretion in CTRL hCS. Finally, our findings further support the involvement of mitochondrial regulation in the mechanisms of action of Li. These results illustrate how patient iPSC-derived 3D hCS can be used as a biological model to provide new insight into BD pathophysiology and to advance our understanding of the cellular mechanisms underlying the therapeutic effects of Li.

### Supplementary information

Supplementary Figures

Supplementary Figure legends

Supplementary Tables

## Data Availability

The data that support the findings of this study are available from the corresponding author upon reasonable request. The data are not publicly available due to national data privacy regulations as they contain information that could compromise research participant privacy and/or consent.
