# Supplementary figures and images for: Transcriptional and functional effects of lithium in bipolar disorder iPSC-derived cortical spheroids
Source: Mol Psychiatry. 2023 Jan 18;28(7):3033–43. doi: 10.1038/s41380-023-01944-0 (PMC10615757; doi:10.1038/s41380-023-01944-0)

Suppl. Fig. 1.

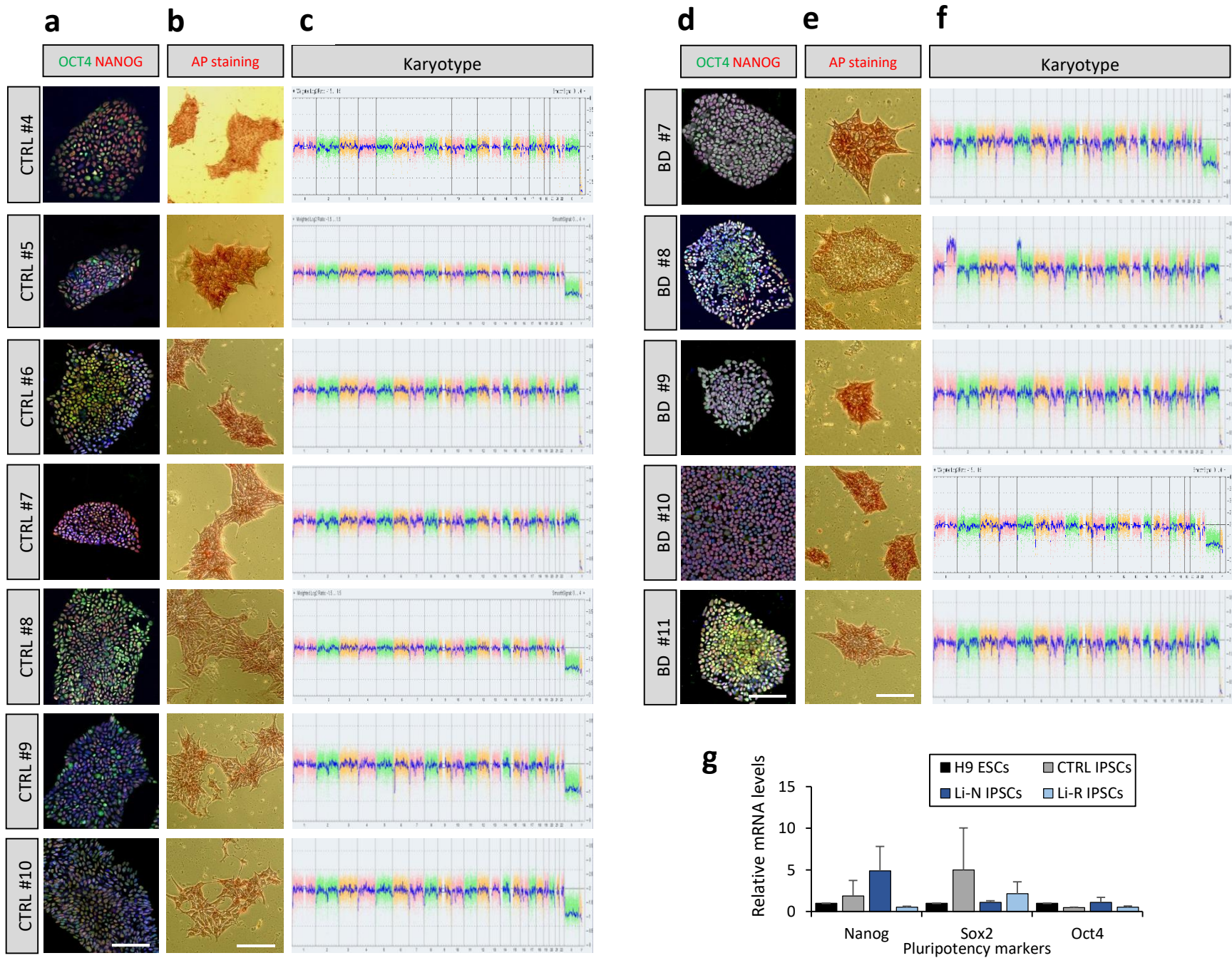

**Suppl. Fig. 2.**

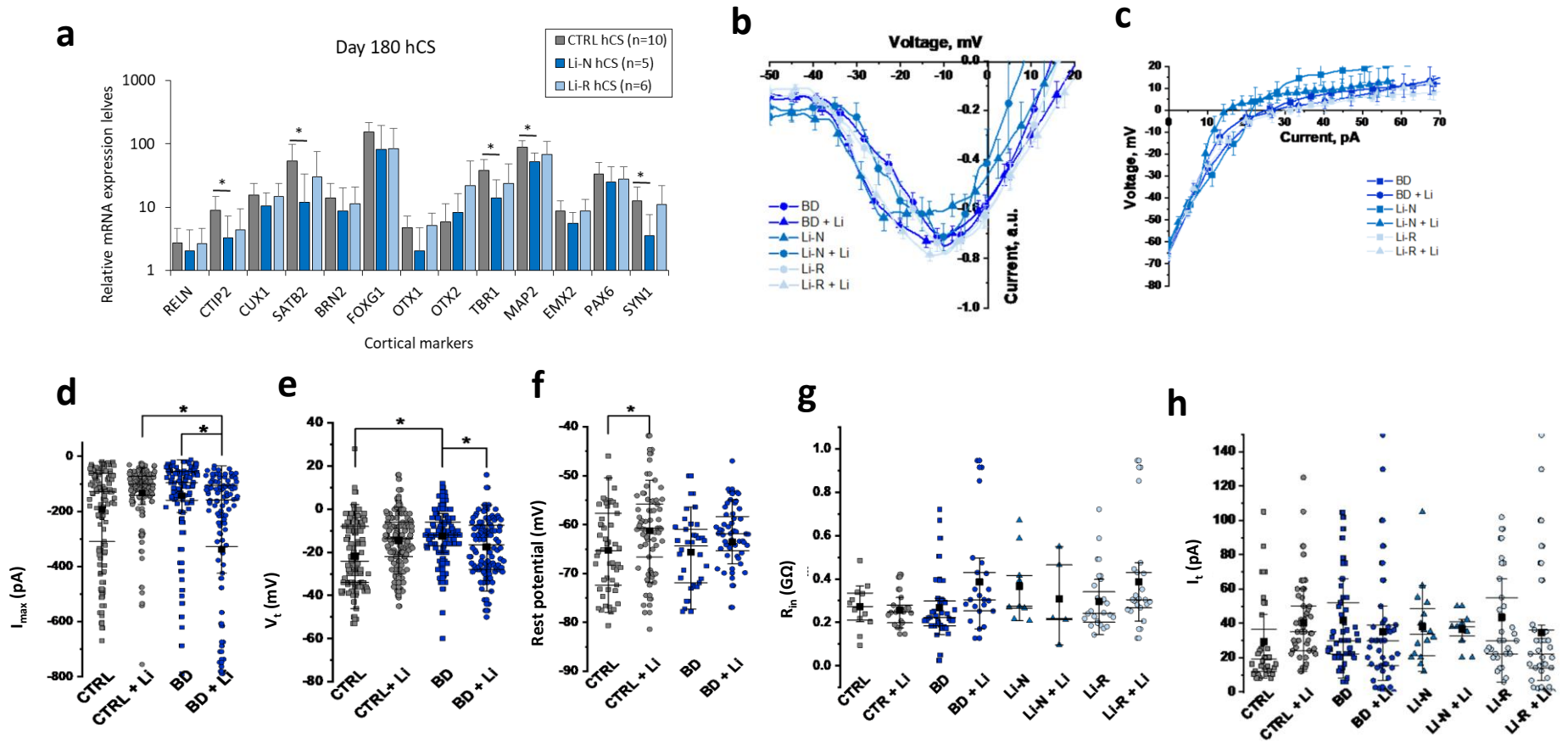

**Suppl. Fig. 3.**

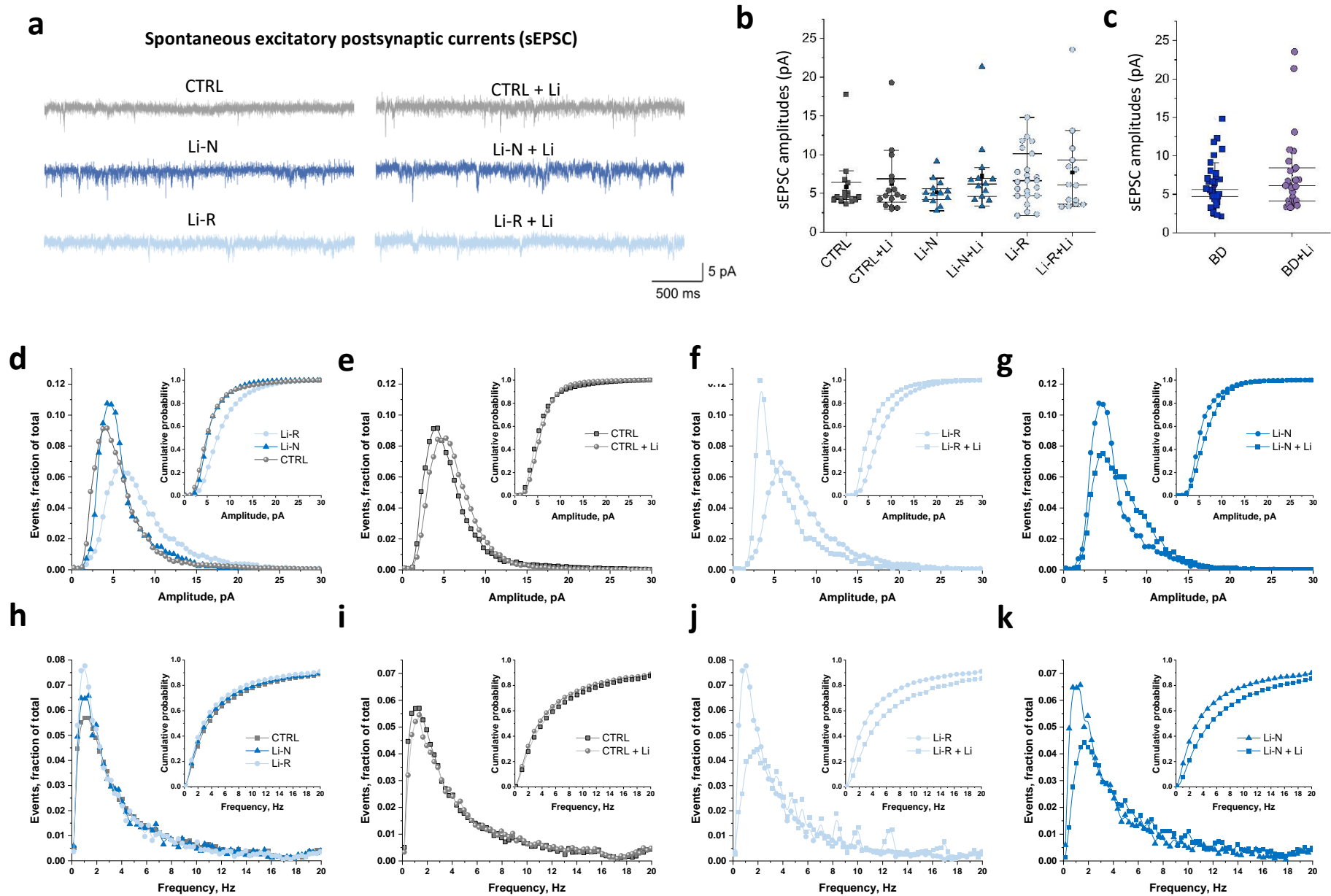

Suppl. Fig. 4.

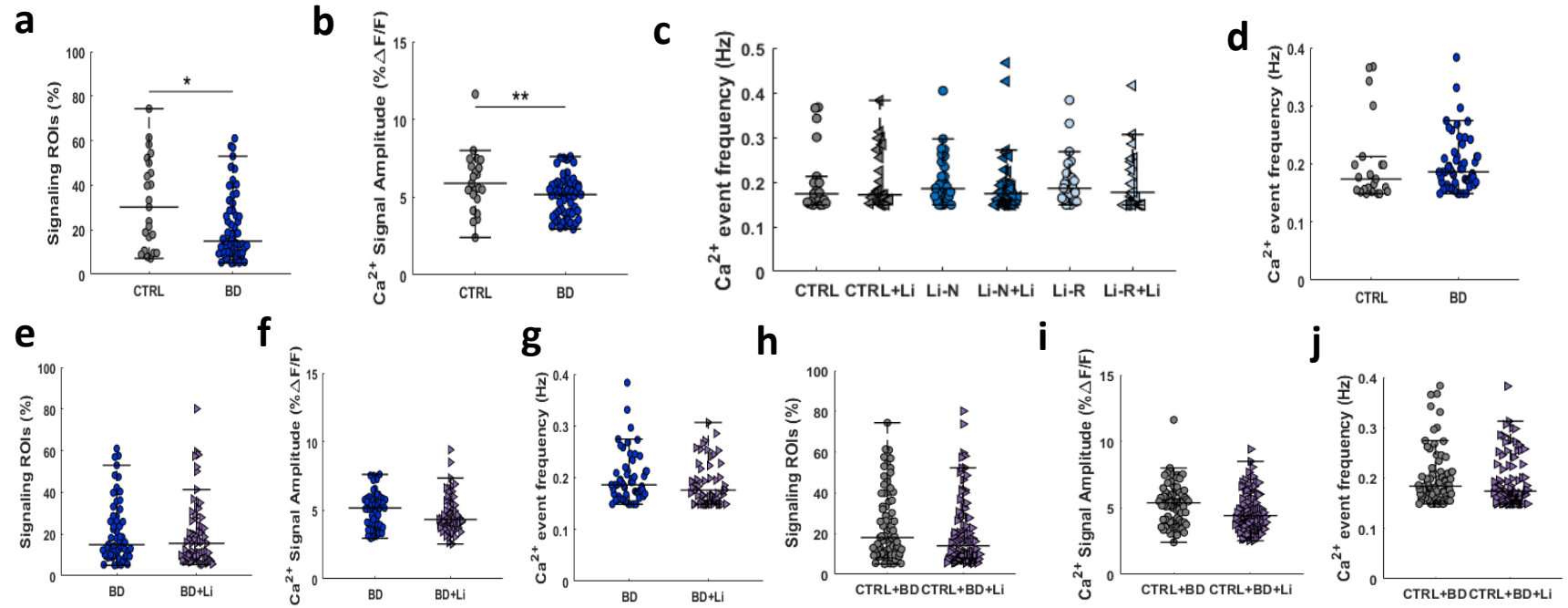

**Suppl. Fig. 5.**

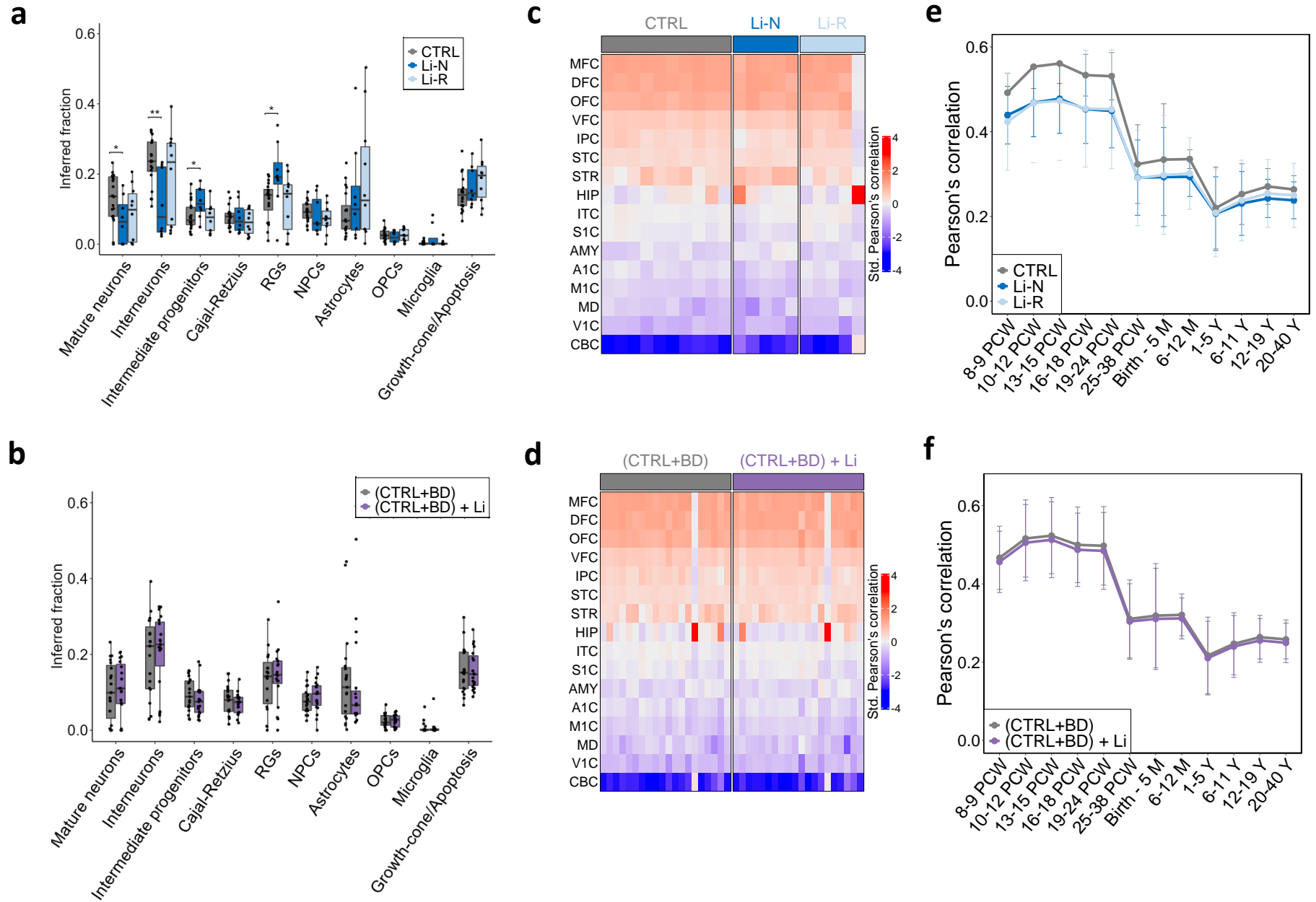

**Suppl. Fig. 6.**

**a**

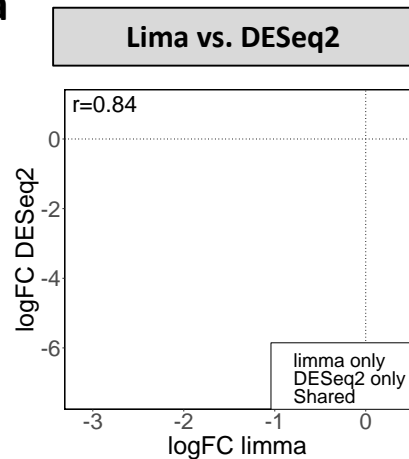

**b**

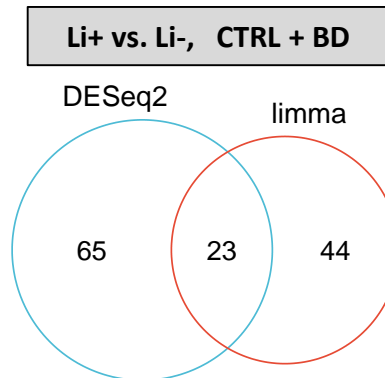

**d**

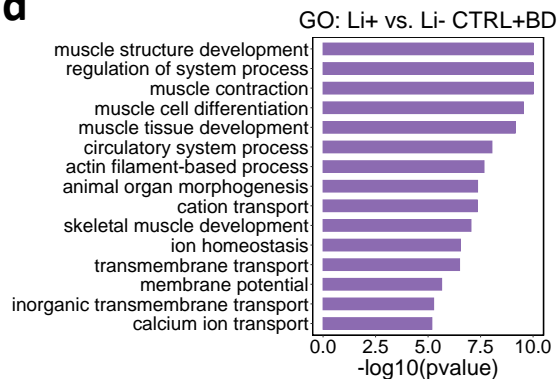

**c**

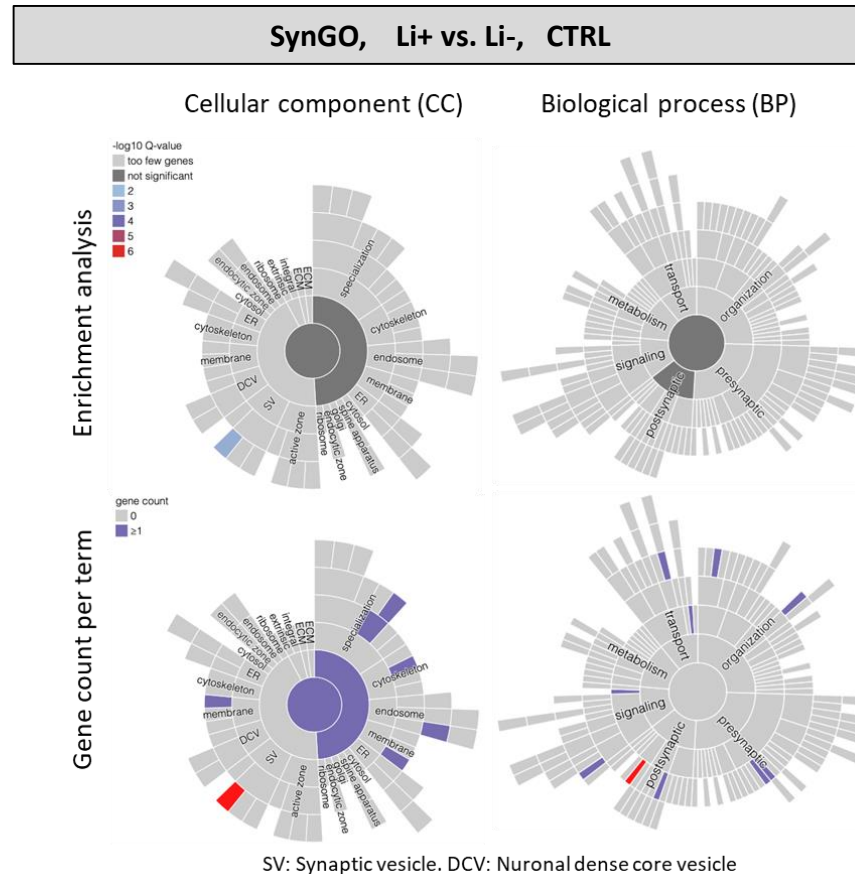

**e**

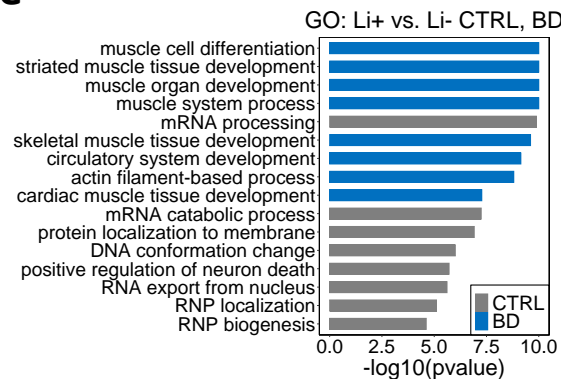

**f**

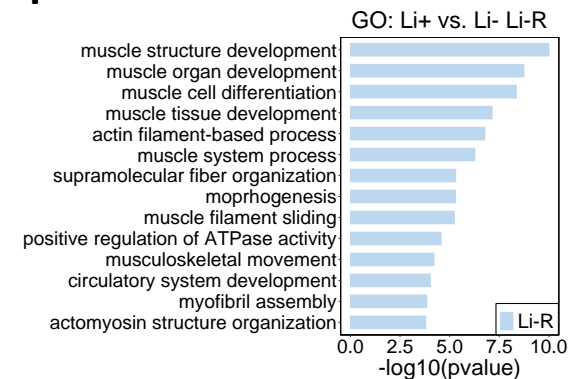

Suppl. Fig. 7.

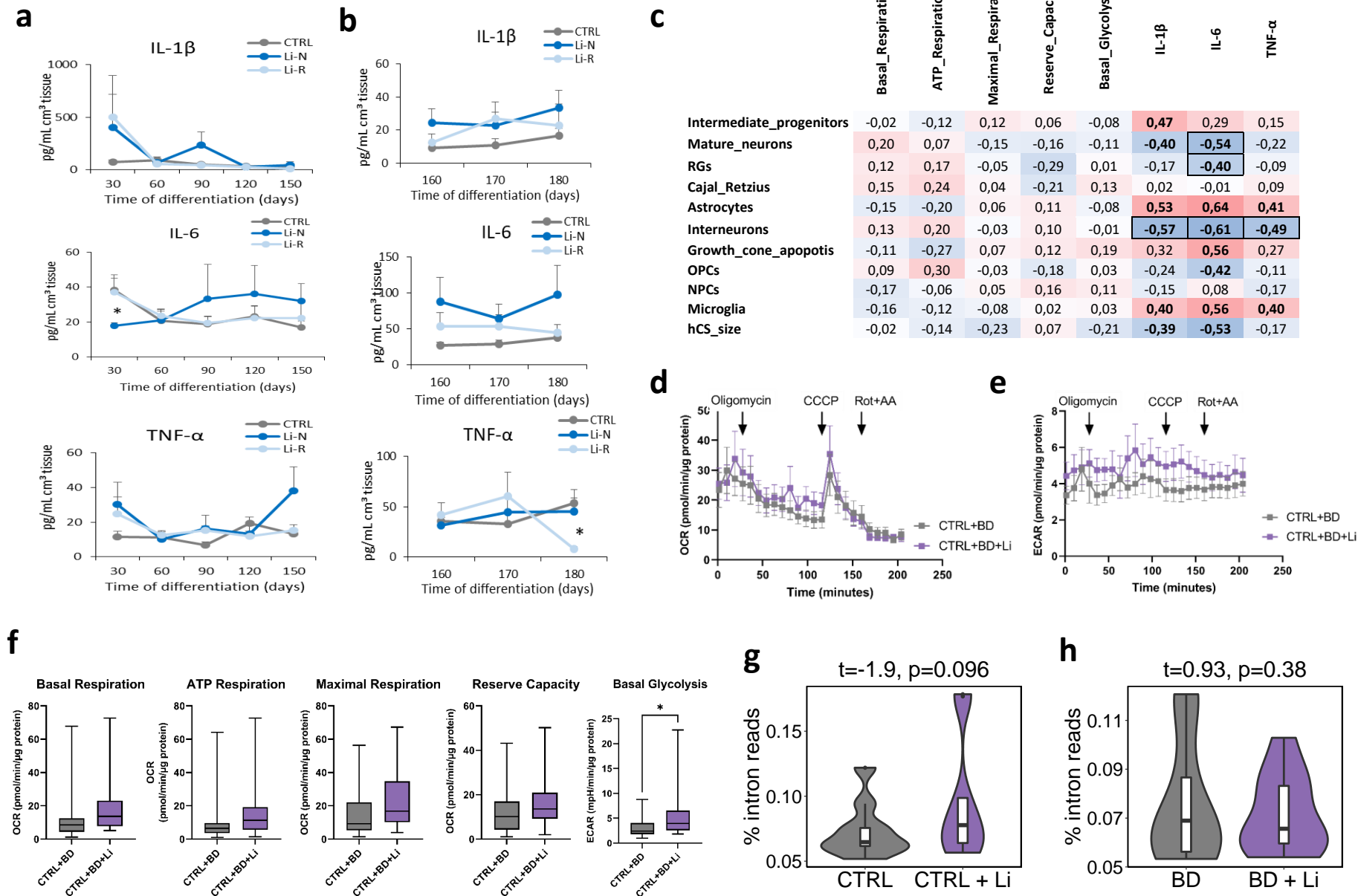

Supplement: Supplementary file 1 — Supplementary Figures [file 41380_2023_1944_MOESM1_ESM.pdf]
