# Supplementary material for: Transcriptional and functional effects of lithium in bipolar disorder iPSC-derived cortical spheroids
Source: Mol Psychiatry. 2023 Jan 18;28(7):3033–43. doi: 10.1038/s41380-023-01944-0 (PMC10615757; doi:10.1038/s41380-023-01944-0)
Supplement: Supplementary file 2 — Supplementary Figure legends [file 41380_2023_1944_MOESM2_ESM.docx]

**SUPPLEMENTARY FIGURE LEGENDS**

**Supplementary Figure 1.**

IPSCs characterization. CTRL#1-3 and BD#1-6 iPSCs were previously characterized (5). **(A, D)** Immunofluorescence staining for each CTRL **(A)** and BD line **(D).** Nuclear Oct4 and Nanog pluripotency markers are shown in the merged image. Scale bars: 100 µm. **(B, E)** Alkaline phosphatase (AP) staining in each CTRL **(B)** and BD line **(E).** Scale bars 100 µm. **(C, F)** Karyotype for each CTRL **(A)** and BD line **(D)**, assessed by KaryoStat™ analysis. Somatic and sex chromosomes are displayed together. The y-axis displays signal intensities in log2-space for microarray probes. A copy number (CN) value of 2 represents a normal copy number state. Chromosomal gains are represented by a value of 3. Chromosomal losses are represented by a value of 1. Pink, green and yellow colours indicate each individual chromosome probe´s raw signal. The blue line represents the normalized probe signal used to identify copy number and aberrations. A near-identical partial chromosomal loss of ~7000 kb was detected in three of the BD lines (#5, #6 and #10) at the 6q24.3 locus. Furthermore, BD#8 contained a 98.7kb and 39.6kb gains in the 1q21.3 and 5p15.31 loci respectively, and CTRL#9 line a 6.97kb loss in the 6p24.3 locus. **(G)** Real time PCR analysis of pluripotency marker genes *NANOG*, *SOX2* and *OCT4* in CTRL and BD iPSCs compared to H9 embryonic stem cells (ESCs). Data is presented as mean ± SD.

**Supplementary Figure 2.**

**(A)** RT-PCR for forebrain development and cortical markers. Data is presented as mean ± SD. Groups were compared by Wilcoxon test. **(B)** Current-voltage (IV) curves for each different BD hCS groupings. **(C)** Voltage-current (VI) curves for each BD hCS groupings. **(D)** Evoked whole-cell currents (I_max_) peak maximum values for CTRL vs. BD conditions. I_max_ distributions analysis showed high variability within and among groups, usually depending on cells’ viability, size and ion channels’ density. **(E)** Threshold voltages (V_t_) values for CTRL vs. BD conditions. **(F)** Membrane resting potential for CTRL (n=14), CTRL + Li (n=22), BD (n=11) and BD + Li (n=17).  **(G)** Input resistance (R_in_) for each condition. **(H)** Threshold currents (I_t_) of action potential induction for each condition. Data was analysed by Mann-Whitney U test and Wilcoxon test for unpaired and paired group comparisons respectively. * P < 0.05.

**Supplementary Figure 3.**

**(A)** Representative traces of whole-cell patch-clamp recordings of spontaneous excitatory postsynaptic currents (sEPSC) for each condition. Data was analyzed from CTRL (10 donors, n=47), CTRL + Li (10 donors, n=42), Li-N (5 donors, n=14), Li-R (5 donors, n=23), Li-N + Li (4 donors, n=13), Li-R + Li (5 donors, n=16). **(B-C)** Averaged sEPSC amplitudes showing no significant differences between conditions. Each dot corresponds to averaged amplitude of the single cell recording. **(D-G)** Distribution histograms of sEPSC amplitudes. **(H-K)** Distribution histograms of sEPSC frequencies showing no differences between conditions. The inset graphs show cumulative probability of sEPSC amplitudes or frequencies for listed samples. No significant differences were found between conditions.

**Supplementary Figure 4.**

**(A)** Percentage of signaling ROIs in untreated CTRL and BD hCS. **(B)** Average amplitude of Ca^2+^ signal transients (%ΔF/F). **(C-D)** Average frequency of Ca^2+^ signal events (Hz) for each condition individually **(C)** and grouped in untreated BD and CTRL hCS **(D).** **(E-G)** Li treatment effect in BD hCS for the percentage of Ca^2+^ signaling ROIs **(E)**, amplitudes **(F)** and frequencies **(G). (H-J)** Li treatment effect in hCS independent of diagnosis (CTRL+BD donors) for the percentage of Ca^2+^ signaling ROIs **(H)**, amplitudes **(I)** and frequencies **(J)**. Data was analysed by Mann-Whitney U test and Wilcoxon test for unpaired and paired group comparisons respectively. * P < 0.05.

**Supplementary Figure 5.**

**(A-B)** Transcriptional deconvolution bar plots showing effects of response status **(A)** and Li treatment **(B)** on brain cell-type composition. **(C-D)** Overlap between *in vitro* gene expression and *in vivo* spatial gene expression profiles (BrainSpan). **(E-F)** Overlap between *in vitro* expression profiles (hCS) and *in vivo* human temporal brain expression profiles. CTRL (N = 10), Li-N (N=5), Li-R (N=6). Kruskal-Wallis and Wilcoxon tests were used for three and two group comparisons of cell fractions. OPCs: oligodendrocyte precursor cells; RGs: radial glia cells; NPCs: neural precursor cells. * P < 0.05.

**Supplementary Figure 6.**

**(A)** High logFC concordance (Pearson’s r) between DEGs identified by limma and DESeq2 in the second DE set. **(B)** Venn diagram showing overlap of DEGs identified by DESeq2 and limma for the DE analysis between Li-treated and untreated hCS (CTRL+BD). **(C)** SynGO analysis of the 74 Li-associated DEGs unique in CTRL hCS, showing genes enriched for *synaptic vesicle*.  **(D-F)** GO pathway enrichment analysis of Li-associated DEGs obtained with GSEA in all samples independent of diagnosis **(D)**, in CTRL and BD hCS separately **(E)**, and in Li-R hCS separately **(F).**

**Supplementary Figure 7.**

**(A-B)** IL-1β, IL-6 and TNF-α cytokine levels in CTRL, Li-N and Li-R hCS at different time points. Data is presented as mean ± SEM. **(C)** Heatmap showing results of a regression model between cell fractions estimated by computational deconvolution and hCS size as dependent variables and mitochondrial parameters and cytokine secretion values paired for each donor. Positive and negative correlations are marked as red and blue gradient colours respectively. Bold numbers indicate significant associations in the partial regression and squares indicate significant associations in the stepwise regression model. **(D-E)** OCR **(D)** and ECAR **(E)** graphs for Li treatment in all donor hCS independent of diagnosis (CTRL+BD). **(F)** Mitochondrial parameters after Li treatment in all hCS. All experiments were run in quadruplicates, and values are normalized to total protein. Data is presented as mean ± SEM. Data was analysed by Mann-Whitney U test and Wilcoxon test for unpaired and paired group comparisons respectively. * P < 0.05. **(G-H)** Li treatment effect on intron retention rate (IRR) in CTRL **(G)** and BD **(H)** hCS. IRR was determined by calculating the proportion of sequencing reads mapping to intronic regions.
