# Supplementary material for: Transcriptional and functional effects of lithium in bipolar disorder iPSC-derived cortical spheroids
Source: Mol Psychiatry. 2023 Jan 18;28(7):3033–43. doi: 10.1038/s41380-023-01944-0 (PMC10615757; doi:10.1038/s41380-023-01944-0)
Supplement: Supplementary file 3 — Supplementary Tables [file 41380_2023_1944_MOESM3_ESM.pdf]

Supplementary Table 1. Patients and healthy controls donor information. CTRL#1 to #3 and BD#1 to #6 were previously characterized (Osete al., 2021).

Donor	Ethnicity	Gender	Age biopsy	Lithium responsive	Type of affective disorder	Clinical data
CTRL#4	European (caucasian)	Female	28	n.a.	n.a.	n.a.
CTRL#5	European (caucasian)	Male	22	n.a.	n.a.	n.a.
CTRL#6	European (caucasian)	Female	56	n.a.	n.a.	n.a.
CTRL#7	European (caucasian)	Female	35	n.a.	n.a.	n.a.
CTRL#8	European (caucasian)	Male	29	n.a.	n.a.	n.a.
CTRL#9	European (caucasian)	Male	19	n.a.	n.a.	n.a.
CTRL#10	European (caucasian)	Male	41	n.a.	n.a.	n.a.
Patient#7	European (caucasian)	Female	46	Li non-treated (Li-N)	Bipolar I disorder (BDI)	Psychotic and manic episodes. Treated with antipsychotics (risperidon) in periods.
Patient#8	European (caucasian)	Female	21	Li non-treated (Li-N)	Bipolar II disorder (BDII)	Several depressive and hypomanic episodes. Treated with antidepressants (mitrazapin) in periods.
Patient#9	European (caucasian)	Female	40	Li responsive (Li-R)	Bipolar I disorder (BDI)	Prescribed Li for 3 years and 3 months. Li daily dose:207.5 mg lithionit. Compliance is assumed to be good and serum level values are unknown. Patient stopped Li due to side-effects, not due to non-response. Several depressive and manic episodes. Treated with antipsychotics (olanzapin) and antidepressants (sertralin) in periods.
Patient#10	European (caucasian)	Male	49	Li responsive (Li-R)	Bipolar I disorder (BDI)	Prescribed Li for 6 months. Li daily dose: 83 mg. Compliance: 100%. Serum levels: 0.56 mmol/L. Prescribed quetiapine, pregabalin and bupropion in periods.
Patient#11	Latin American	Male	38	Li responsive (Li-R)	Bipolar NOS / Schizophrenia residual type	Prescribed Li for 10 months. Li daily dose: 166 mg. Compliance: 100%. Serum levels: 0.68 mmol/L. Prescribed quetiapine and valproate in periods.

Supplementary Table 2. RT-PCR primers used for iPSC characterization.

Gene	Forward primer	Reverse primer
OCT4	5’-GTACTCCTCGGTCCCTTTCC-3’	5’-CAAAAACCCTGGCACAAACT-3’
NANOG	5’-AATACCTCAGCCTCCAGCAGATG-3’	5’-TGCGTCACACCATTGCTATTCTTC-3’
SOX2	5’-TGCGAGCGCTGCACAT-3’	5’-TCATGAGCGTCTTGGTTTTCC-3’
GAPDH	5’-GTCAACGGATTTGGTCGTATTG-3’	5’-CATGGGTGGAATCATATTGGAA-3’

Supplementary Table 3. For cortical spheroids (hCS) characterization, a custom gene expression TaqMan array card was used, containing primers for the detection of the following genes.

Gene Symbol	Gene Name	Context Sequence	Assay ID
ACTB	actin beta	CCTTTGCCGATCCGCCGCCCGTCCA	Hs99999903_m1
RELN	reelin	CAATGTCCCCCTGGAGGCACGGATG	Hs01022646_m1
BCL11B	B-cell CLL/lymphoma 11B	GAACATTGCAGGTAAAGATGAGCCT	Hs01102259_m1
GAPDH	glyceraldehyde-3-phosphate dehydrogenase	GGGCGCCTGGTCACCAGGGCTGCTT	Hs99999905_m1
CUX1	cut like homeobox 1	AACAGCACACTCAAACAACTGGAAG	Hs00738851_m1
SATB2	SATB homeobox 2	TAAAAGTCAGGGCTGGCTGTGTGAA	Hs01546836_m1
POU3F2	POU class 3 homeobox 2	GTGTTTTGACCTTTGCAGGCGAGTA	Hs00271595_s1
FOXG1	forkhead box G1	CATCAACAGCCTGGTGCCCGAGGCG	Hs01850784_s1
OTX1	orthodenticle homeobox 1	TGGGCTATCCGGCCACTCCGCGGAA	Hs00951099_m1
OTX2	orthodenticle homeobox 2	CCGAGTCGAGGGTGCAGGTATGGTT	Hs00222238_m1
TBR1	T-box, brain 1	GATAATTATGACACGATCTACACCG	Hs00232429_m1
MAP2	microtubule associated protein 2	GCCACAGGCCAGGTGGCGGACGTGT	Hs00258900_m1
EMX2	empty spiracles homeobox 2	TCACGGAAACTCAGGTAAAAGTATG	Hs00244574_m1
PAX6	paired box 6	CAACCTACGCAAGATGGCTGCCAGC	Hs01088114_m1
SYN1	synapsin I	CAAAGAAATGCTCAGCAGTACAACG	Hs00199577_m1
HMBS	hydroxymethylbilane synthase	ATGCGGCTGCAACGGCGGAAGAAAA	Hs00609297_m1

Supplementary Table 4. Primary and secondary antibodies used for IF and IHC.

Antibody	Species	Technique	Dilution	Company	Reference
Oct4	mouse	IF	1:200	Stem Cell Technologies	60093.1
Nanog	rabbit	IF	1:200	Stemgent	09-0020
Map2	chicken	IHC	1:10000	Abcam	ab5392
SV2A	rabbit	IHC	1:400	Cell Signaling technologies	66724
Anti-rabbit Alexa 488	goat	IF / IHC	1:200	Invitrogen	A32731
Anti-mouse Alexa 594	donkey	IF	1:200	Invitrogen	A21203
Anti-chicken Alexa 647	goat	IHC	1:200	Invitrogen	A21449

Supplementary Table 5. Genes excluded from the DE analysis, based on karyostat results.

ensembl_gene_id	external_gene_name	gene_biotype	chromosome	start_position	end_position	strand
ENSG00000263513	FAM72C	protein_coding	1	143955287	143971986	-1
ENSG00000143384	MCL1	protein_coding	1	150560895	150579738	-1
ENSG00000143420	ENSA	protein_coding	1	150600851	150629612	-1
ENSG00000143457	GOLPH3L	protein_coding	1	150646230	150697154	-1
ENSG00000163131	CTSS	protein_coding	1	150730079	150765957	-1
ENSG00000143387	CTSK	protein_coding	1	150794880	150809577	-1
ENSG00000143437	ARNT	protein_coding	1	150809713	150876708	-1
ENSG00000143379	SETDB1	protein_coding	1	150926263	150964744	1
ENSG00000143418	CERS2	protein_coding	1	150960583	150975003	-1
ENSG00000143409	MINDY1	protein_coding	1	150996549	151008376	-1
ENSG00000143363	PRUNE1	protein_coding	1	151008420	151035713	1
ENSG00000163141	BNIPL	protein_coding	1	151036321	151047720	1
ENSG00000143443	C1orf56	protein_coding	1	151047751	151051986	1
ENSG00000197622	CDC42SE1	protein_coding	1	151050971	151070325	-1
ENSG00000213190	MLLT11	protein_coding	1	151060397	151069544	1
ENSG00000143458	GABPB2	protein_coding	1	151070578	151125542	1
ENSG00000143434	SEMA6C	protein_coding	1	151131685	151146631	-1
ENSG00000163156	SCNM1	protein_coding	1	151156664	151170296	1
ENSG00000163155	LYSMD1	protein_coding	1	151159748	151165948	-1
ENSG00000163157	TMOD4	protein_coding	1	151169986	151176284	-1
ENSG00000163159	VPS72	protein_coding	1	151176304	151195321	-1
ENSG00000143398	PIP5K1A	protein_coding	1	151197949	151249536	1
ENSG00000159352	PSMD4	protein_coding	1	151254709	151267479	1
ENSG00000143373	ZNF687	protein_coding	1	151281618	151292176	1
ENSG00000143393	PI4KB	protein_coding	1	151291797	151327715	-1
ENSG00000143390	RFX5	protein_coding	1	151340640	151347326	-1
ENSG00000143416	SELENBP1	protein_coding	1	151364304	151372707	-1
ENSG00000159377	PSMB4	protein_coding	1	151399560	151401937	1
ENSG00000143442	POGZ	protein_coding	1	151402724	151459494	-1
ENSG00000143375	CGN	protein_coding	1	151510510	151538692	1
ENSG00000143367	TUFT1	protein_coding	1	151540305	151583583	1
ENSG00000143376	SNX27	protein_coding	1	151612006	151699091	1
ENSG00000159409	CELF3	protein_coding	1	151700058	151716803	-1
ENSG00000178796	RIIAD1	protein_coding	1	151710433	151729805	1
ENSG00000143436	MRPL9	protein_coding	1	151759647	151763496	-1
ENSG00000143450	OAZ3	protein_coding	1	151762899	151771334	1
ENSG00000182134	TDRKH	protein_coding	1	151770107	151791416	-1
ENSG00000143365	RORC	protein_coding	1	151806071	151831845	-1
ENSG00000159445	THEM4	protein_coding	1	151870866	151909637	-1
ENSG00000197747	S100A10	protein_coding	1	151982915	151993859	-1
ENSG00000163191	S100A11	protein_coding	1	152032506	152047907	-1
ENSG00000197956	S100A6	protein_coding	1	153534599	153536244	-1
ENSG00000196154	S100A4	protein_coding	1	153543613	153550136	-1
ENSG00000196754	S100A2	protein_coding	1	153561108	153567890	-1
ENSG00000188643	S100A16	protein_coding	1	153606886	153613145	-1
ENSG00000189171	S100A13	protein_coding	1	153618787	153631360	-1
ENSG00000160678	S100A1	protein_coding	1	153627926	153632039	1
ENSG00000160679	CHTOP	protein_coding	1	153633982	153646306	1
ENSG00000143553	SNAPIN	protein_coding	1	153658703	153661852	1
ENSG00000143621	ILF2	protein_coding	1	153661788	153671028	-1
ENSG00000169418	NPR1	protein_coding	1	153678688	153693992	1
ENSG00000143624	INTS3	protein_coding	1	153728050	153774808	1
ENSG00000143554	SLC27A3	protein_coding	1	153774354	153780157	1
ENSG00000143614	GATAD2B	protein_coding	1	153789030	153923360	-1
ENSG00000198837	DENND4B	protein_coding	1	153929501	153946718	-1
ENSG00000160741	CRTC2	protein_coding	1	153947669	153958615	-1
ENSG00000143570	SLC39A1	protein_coding	1	153959099	153968184	-1
ENSG00000143578	CREB3L4	protein_coding	1	153967534	153974361	1
ENSG00000143543	JTB	protein_coding	1	153974269	153977674	-1

ENSG00000143545	RAB13	protein_coding	1	153981617	153986358	-1
ENSG00000177954	RPS27	protein_coding	1	153990762	153992155	1
ENSG00000143549	TPM3	protein_coding	1	154155304	154194648	-1
ENSG00000163263	C1orf189	protein_coding	1	154199085	154206333	-1
ENSG00000143612	C1orf43	protein_coding	1	154206696	154220637	-1
ENSG00000143569	UBAP2L	protein_coding	1	154220179	154271510	1
ENSG00000143575	HAX1	protein_coding	1	154272589	154275875	1
ENSG00000143515	ATP8B2	protein_coding	1	154325525	154351304	1
ENSG00000160712	IL6R	protein_coding	1	154405193	154469450	1
ENSG00000169291	SHE	protein_coding	1	154469772	154502412	-1
ENSG00000160714	UBE2Q1	protein_coding	1	154548577	154559028	-1
ENSG00000160716	CHRNB2	protein_coding	1	154567778	154580013	1
ENSG00000160710	ADAR	protein_coding	1	154581695	154628013	-1
ENSG00000143603	KCNN3	protein_coding	1	154697455	154870281	-1
ENSG00000163344	PMVK	protein_coding	1	154924740	154936719	-1
ENSG00000163346	PBXIP1	protein_coding	1	154944076	154956123	-1
ENSG00000163348	PYGO2	protein_coding	1	154957026	154963853	-1
ENSG00000160691	SHC1	protein_coding	1	154962298	154974395	-1
ENSG00000173207	CKS1B	protein_coding	1	154974653	154979251	1
ENSG00000160688	FLAD1	protein_coding	1	154983338	154993111	1
ENSG00000163352	LENEP	protein_coding	1	154993586	154994315	1
ENSG00000160685	ZBTB7B	protein_coding	1	155002630	155018523	1
ENSG00000163354	DCST2	protein_coding	1	155018520	155033781	-1
ENSG00000143537	ADAM15	protein_coding	1	155050566	155062775	1
ENSG00000243364	EFNA4	protein_coding	1	155063737	155069553	1
ENSG00000143590	EFNA3	protein_coding	1	155078837	155087538	1
ENSG00000169242	EFNA1	protein_coding	1	155127876	155134899	1
ENSG00000169241	SLC50A1	protein_coding	1	155135344	155138857	1
ENSG00000179085	DPM3	protein_coding	1	155139891	155140595	-1
ENSG00000163463	KRTCAP2	protein_coding	1	155169408	155173475	-1
ENSG00000163462	TRIM46	protein_coding	1	155173787	155184971	1
ENSG00000185499	MUC1	protein_coding	1	155185824	155192916	-1
ENSG00000169231	THBS3	protein_coding	1	155195588	155209051	-1
ENSG00000173171	MTX1	protein_coding	1	155208695	155213824	1
ENSG00000177628	GBA	protein_coding	1	155234452	155244699	-1
ENSG00000160767	FAM189B	protein_coding	1	155247205	155255483	-1
ENSG00000116521	SCAMP3	protein_coding	1	155255979	155262430	-1
ENSG00000176444	CLK2	protein_coding	1	155262868	155278491	-1
ENSG00000143630	HCN3	protein_coding	1	155277463	155289848	1
ENSG00000160752	FDPS	protein_coding	1	155308748	155320666	1
ENSG00000160753	RUSC1	protein_coding	1	155320894	155331114	1
ENSG00000116539	ASH1L	protein_coding	1	155335268	155563162	-1
ENSG00000125459	MSTO1	protein_coding	1	155610205	155614951	1
ENSG00000163374	YY1AP1	protein_coding	1	155659443	155689000	-1
ENSG00000132676	DAP3	protein_coding	1	155687960	155739010	1
ENSG00000116580	GON4L	protein_coding	1	155749659	155859400	-1
ENSG00000132718	SYT11	protein_coding	1	155859567	155885199	1
ENSG00000143622	RIT1	protein_coding	1	155897808	155911404	-1
ENSG00000132680	KHDC4	protein_coding	1	155913045	155934413	-1
ENSG00000116584	ARHGEF2	protein_coding	1	155946851	156007070	-1
ENSG00000163479	SSR2	protein_coding	1	156009048	156020951	-1
ENSG00000160803	UBQLN4	protein_coding	1	156035299	156053798	-1
ENSG00000116586	LAMTOR2	protein_coding	1	156054782	156058506	1
ENSG00000254726	MEX3A	protein_coding	1	156072013	156082465	-1
ENSG00000160789	LMNA	protein_coding	1	156082573	156140081	1
ENSG00000196189	SEMA4A	protein_coding	1	156147366	156177752	1
ENSG00000160785	SLC25A44	protein_coding	1	156193932	156212796	1
ENSG00000160783	PMF1	protein_coding	1	156212993	156240042	1
ENSG00000160781	PAQR6	protein_coding	1	156243320	156248117	-1
ENSG00000198952	SMG5	protein_coding	1	156249224	156282825	-1
ENSG00000163472	TMEM79	protein_coding	1	156282935	156293185	1
ENSG00000198715	GLMP	protein_coding	1	156290089	156295689	-1
ENSG00000163468	CCT3	protein_coding	1	156308968	156367873	-1

ENSG00000163467	TSACC	protein_coding	1	156337314	156346995	1
ENSG00000116604	MEF2D	protein_coding	1	156463727	156500779	-1
ENSG00000183856	IQGAP3	protein_coding	1	156525405	156572604	-1
ENSG00000163382	NAXE	protein_coding	1	156591756	156609507	1
ENSG00000160818	GPATCH4	protein_coding	1	156594301	156601496	-1
ENSG00000132702	HAPLN2	protein_coding	1	156619331	156625725	1
ENSG00000132692	BCAN	protein_coding	1	156641390	156659532	1
ENSG00000132688	NES	protein_coding	1	156668763	156677407	-1
ENSG00000143320	CRABP2	protein_coding	1	156699606	156705816	-1
ENSG00000143319	ISG20L2	protein_coding	1	156721891	156728766	-1
ENSG00000143314	MRPL24	protein_coding	1	156737303	156741590	-1
ENSG00000143321	HDGF	protein_coding	1	156742109	156766925	-1
ENSG00000143294	PRCC	protein_coding	1	156750610	156800815	1
ENSG00000132694	ARHGEF11	protein_coding	1	156934840	157045742	-1
ENSG00000117036	ETV3	protein_coding	1	157121191	157138474	-1
ENSG00000160856	FCRL3	protein_coding	1	157674321	157700769	-1
ENSG00000183853	KIRREL1	protein_coding	1	157993273	158100262	1
ENSG00000163565	IFI16	protein_coding	1	158999968	159055155	1
ENSG00000163568	AIM2	protein_coding	1	159062484	159147096	-1
ENSG00000162706	CADM3	protein_coding	1	159171609	159203313	1
ENSG00000213088	ACKR1	protein_coding	1	159203307	159206500	1
ENSG00000158716	DUSP23	protein_coding	1	159780932	159782543	1
ENSG00000213085	CFAP45	protein_coding	1	159872364	159900165	-1
ENSG00000158710	TAGLN2	protein_coding	1	159918107	159925507	-1
ENSG00000085552	IGSF9	protein_coding	1	159927039	159945613	-1
ENSG00000177807	KCNJ10	protein_coding	1	159998651	160070160	-1
ENSG00000143315	PIGM	protein_coding	1	160024953	160031990	-1
ENSG00000162728	KCNJ9	protein_coding	1	160081538	160090563	1
ENSG00000162729	IGSF8	protein_coding	1	160091340	160098943	-1
ENSG00000018625	ATP1A2	protein_coding	1	160115759	160143591	1
ENSG00000132681	ATP1A4	protein_coding	1	160151586	160186980	1
ENSG00000143318	CASQ1	protein_coding	1	160190575	160201886	1
ENSG00000162734	PEA15	protein_coding	1	160205380	160215376	1
ENSG00000132716	DCAF8	protein_coding	1	160215715	160262549	-1
ENSG00000162735	PEX19	protein_coding	1	160276807	160286348	-1
ENSG00000122218	COPA	protein_coding	1	160288594	160343273	-1
ENSG00000162736	NCSTN	protein_coding	1	160343316	160358952	1
ENSG00000171786	NHLH1	protein_coding	1	160367071	160372846	1
ENSG00000162738	VANGL2	protein_coding	1	160400564	160428670	1
ENSG00000158769	F11R	protein_coding	1	160995211	161021343	-1
ENSG00000215845	TSTD1	protein_coding	1	161037631	161038977	-1
ENSG00000158773	USF1	protein_coding	1	161039251	161045977	-1
ENSG00000143217	NECTIN4	protein_coding	1	161070998	161089558	-1
ENSG00000162755	KLHDC9	protein_coding	1	161098361	161100346	1
ENSG00000143256	PFDN2	protein_coding	1	161100556	161118055	-1
ENSG00000158793	NIT1	protein_coding	1	161118086	161125445	1
ENSG00000158796	DEDD	protein_coding	1	161120974	161132688	-1
ENSG00000143222	UFC1	protein_coding	1	161152776	161158856	1
ENSG00000143258	USP21	protein_coding	1	161159450	161165723	1
ENSG00000143224	PPOX	protein_coding	1	161166056	161178013	1
ENSG00000158850	B4GALT3	protein_coding	1	161171310	161177968	-1
ENSG00000158859	ADAMTS4	protein_coding	1	161184302	161199054	-1
ENSG00000158864	NDUFS2	protein_coding	1	161197104	161214723	1
ENSG00000158869	FCER1G	protein_coding	1	161215234	161220699	1
ENSG00000158882	TOMM40L	protein_coding	1	161225939	161230746	1
ENSG00000248485	PCP4L1	protein_coding	1	161258745	161285450	1
ENSG00000158887	MPZ	protein_coding	1	161304735	161309968	-1
ENSG00000143252	SDHC	protein_coding	1	161314381	161363206	1
ENSG00000188931	CFAP126	protein_coding	1	161364733	161367876	-1
ENSG00000143226	FCGR2A	protein_coding	1	161505430	161524013	1
ENSG00000173110	HSPA6	protein_coding	1	161524540	161526894	1
ENSG00000132185	FCRLA	protein_coding	1	161706972	161714352	1
ENSG00000162746	FCRLB	protein_coding	1	161721563	161728143	1

ENSG00000081721	DUSP12	protein_coding	1	161749758	161757238	1
ENSG00000118217	ATF6	protein_coding	1	161766298	161977574	1
ENSG00000162745	OLFML2B	protein_coding	1	161983192	162023869	-1
ENSG00000198929	NOS1AP	protein_coding	1	162069691	162370475	1
ENSG00000239887	C1orf226	protein_coding	1	162378841	162386812	1
ENSG00000152332	UHMK1	protein_coding	1	162497251	162529631	1
ENSG00000117143	UAP1	protein_coding	1	162561506	162599842	1
ENSG00000162733	DDR2	protein_coding	1	162631373	162787405	1
ENSG00000132196	HSD17B7	protein_coding	1	162790702	162812823	1
ENSG00000185860	CCDC190	protein_coding	1	162824458	162868761	-1
ENSG00000117152	RGS4	protein_coding	1	163068775	163076802	1
ENSG00000143248	RGS5	protein_coding	1	163111121	163321791	-1
ENSG00000143228	NUF2	protein_coding	1	163266576	163355764	1
ENSG00000185630	PBX1	protein_coding	1	164555584	164899296	1
ENSG00000162761	LMX1A	protein_coding	1	165201867	165356715	-1
ENSG00000143171	RXRG	protein_coding	1	165400922	165445355	-1
ENSG00000143198	MGST3	protein_coding	1	165631213	165661796	1
ENSG00000143149	ALDH9A1	protein_coding	1	165662216	165698562	-1
ENSG00000143183	TMCO1	protein_coding	1	165724293	165827755	-1
ENSG00000143179	UCK2	protein_coding	1	165827614	165911618	1
ENSG00000188859	FAM78B	protein_coding	1	166057426	166167001	-1
ENSG00000143157	POGK	protein_coding	1	166839447	166856359	1
ENSG00000152382	TADA1	protein_coding	1	166856510	166876264	-1
ENSG00000143195	ILDR2	protein_coding	1	166895711	166975540	-1
ENSG00000143194	MAEL	protein_coding	1	166975582	167022214	1
ENSG00000198842	STYXL2	protein_coding	1	167094075	167129165	1
ENSG00000143190	POU2F1	protein_coding	1	167220876	167427345	1
ENSG00000198821	CD247	protein_coding	1	167430640	167518610	-1
ENSG00000143162	CREG1	protein_coding	1	167529117	167553805	-1
ENSG00000198771	RCSD1	protein_coding	1	167630093	167708696	1
ENSG00000197965	MPZL1	protein_coding	1	167721192	167791919	1
ENSG00000143199	ADCY10	protein_coding	1	167809386	167914215	-1
ENSG00000143158	MPC2	protein_coding	1	167916675	167937072	-1
ENSG00000143164	DCAF6	protein_coding	1	167935783	168075843	1
ENSG00000143147	GPR161	protein_coding	1	168079542	168137667	-1
ENSG00000143155	TIPRL	protein_coding	1	168178962	168202109	1
ENSG00000213064	SFT2D2	protein_coding	1	168225938	168253021	1
ENSG00000143178	TBX19	protein_coding	1	168280877	168314426	1
ENSG00000143196	DPT	protein_coding	1	168695468	168729206	-1
ENSG00000143153	ATP1B1	protein_coding	1	169105697	169310992	1
ENSG00000143156	NME7	protein_coding	1	169132531	169367948	-1
ENSG00000117475	BLZF1	protein_coding	1	169367970	169396540	1
ENSG00000117477	CCDC181	protein_coding	1	169394870	169460669	-1
ENSG00000117479	SLC19A2	protein_coding	1	169463909	169485944	-1
ENSG00000198734	F5	protein_coding	1	169511951	169586588	-1
ENSG00000000460	C1orf112	protein_coding	1	169662007	169854080	1
ENSG00000188404	SELL	protein_coding	1	169690665	169711702	-1
ENSG00000171806	METTL18	protein_coding	1	169792529	169794963	-1
ENSG00000000457	SCYL3	protein_coding	1	169849631	169894267	-1
ENSG00000075945	KIFAP3	protein_coding	1	169921326	170085208	-1
ENSG00000120370	GORAB	protein_coding	1	170531819	170553834	1
ENSG00000116132	PRRX1	protein_coding	1	170662728	170739421	1
ENSG00000094963	FMO2	protein_coding	1	171185249	171212686	1
ENSG00000010932	FMO1	protein_coding	1	171248471	171285978	1
ENSG00000076258	FMO4	protein_coding	1	171314183	171342084	1
ENSG00000117523	PRRC2C	protein_coding	1	171485530	171593511	1
ENSG00000117533	VAMP4	protein_coding	1	171700160	171742074	-1
ENSG00000197959	DNM3	protein_coding	1	171817887	172418466	1
ENSG00000135845	PIGC	protein_coding	1	172370189	172444086	-1
ENSG00000094975	SUCO	protein_coding	1	172532349	172611833	1
ENSG00000120337	TNFSF18	protein_coding	1	173039202	173050941	-1
ENSG00000117586	TNFSF4	protein_coding	1	173183731	173207331	-1
ENSG00000117592	PRDX6	protein_coding	1	173477330	173488815	1

ENSG00000162753	SLC9C2	protein_coding	1	173500460	173603072	-1
ENSG00000183831	ANKRD45	protein_coding	1	173608336	173669851	-1
ENSG00000076321	KLHL20	protein_coding	1	173714941	173786692	1
ENSG00000120334	CENPL	protein_coding	1	173799550	173824883	-1
ENSG00000117593	DARS2	protein_coding	1	173824653	173858808	1
ENSG00000185278	ZBTB37	protein_coding	1	173868082	173903549	1
ENSG00000135870	RC3H1	protein_coding	1	173931084	174022357	-1
ENSG00000152061	RABGAP1L	protein_coding	1	174159410	174995308	1
ENSG00000203737	GPR52	protein_coding	1	174447964	174449545	1
ENSG00000116161	CACYBP	protein_coding	1	174999163	175012027	1
ENSG00000120333	MRPS14	protein_coding	1	175010789	175023425	-1
ENSG00000120332	TNN	protein_coding	1	175067833	175148075	1
ENSG00000235750	KIAA0040	protein_coding	1	175156986	175192999	-1
ENSG00000116147	TNR	protein_coding	1	175315194	175743616	-1
ENSG00000143207	COP1	protein_coding	1	175944831	176207286	-1
ENSG00000116183	PAPPA2	protein_coding	1	176463171	176845601	1
ENSG00000152092	ASTN1	protein_coding	1	176857302	177164973	-1
ENSG00000198797	BRINP2	protein_coding	1	177170958	177282422	1
ENSG00000075391	RASAL2	protein_coding	1	178094104	178484147	1
ENSG00000116191	RALGPS2	protein_coding	1	178725165	178921842	1
ENSG00000116194	ANGPTL1	protein_coding	1	178849535	178871077	-1
ENSG00000116199	FAM20B	protein_coding	1	179025804	179076567	1
ENSG00000186283	TOR3A	protein_coding	1	179082070	179098023	1
ENSG00000143322	ABL2	protein_coding	1	179099330	179229684	-1
ENSG00000057252	SOAT1	protein_coding	1	179293714	179358680	1
ENSG00000162779	AXDND1	protein_coding	1	179365720	179554735	1
ENSG00000162782	TDRD5	protein_coding	1	179591613	179691272	1
ENSG00000143340	FAM163A	protein_coding	1	179743291	179816198	1
ENSG00000169905	TOR1AIP2	protein_coding	1	179839967	179877803	-1
ENSG00000143337	TOR1AIP1	protein_coding	1	179882042	179920077	1
ENSG00000135837	CEP350	protein_coding	1	179954773	180114875	1
ENSG00000116260	QSOX1	protein_coding	1	180154869	180204030	1
ENSG00000121454	LHX4	protein_coding	1	180230264	180278984	1
ENSG00000230124	ACBD6	protein_coding	1	180269653	180502954	-1
ENSG00000143324	XPR1	protein_coding	1	180632022	180890279	1
ENSG00000135835	KIAA1614	protein_coding	1	180912897	180951614	1
ENSG00000135823	STX6	protein_coding	1	180972712	181023121	-1
ENSG00000153029	MR1	protein_coding	1	181033374	181061938	1
ENSG00000162783	IER5	protein_coding	1	181088700	181092900	1
ENSG00000198216	CACNA1E	protein_coding	1	181317690	181808084	1
ENSG00000179930	ZNF648	protein_coding	1	182054570	182061712	-1
ENSG00000135821	GLUL	protein_coding	1	182378098	182392206	-1
ENSG00000135828	RNASEL	protein_coding	1	182573634	182589256	-1
ENSG00000143333	RGS16	protein_coding	1	182598623	182604389	-1
ENSG00000135824	RGS8	protein_coding	1	182641816	182684576	-1
ENSG00000135838	NPL	protein_coding	1	182789293	182830384	1
ENSG00000135829	DHX9	protein_coding	1	182839347	182887982	1
ENSG00000135862	LAMC1	protein_coding	1	183023420	183145592	1
ENSG00000058085	LAMC2	protein_coding	1	183186238	183245127	1
ENSG00000157064	NMNAT2	protein_coding	1	183248237	183418380	-1
ENSG00000116698	SMG7	protein_coding	1	183472216	183598246	1
ENSG00000116701	NCF2	protein_coding	1	183555562	183590876	-1
ENSG00000162704	ARPC5	protein_coding	1	183620846	183635783	-1
ENSG00000143344	RGL1	protein_coding	1	183636085	183928532	1
ENSG00000198756	COLGALT2	protein_coding	1	183929854	184037729	-1
ENSG00000198860	TSEN15	protein_coding	1	184051651	184123978	1
ENSG00000116667	C1orf21	protein_coding	1	184387029	184629019	1
ENSG00000116406	EDEM3	protein_coding	1	184690237	184754907	-1
ENSG00000135842	NIBAN1	protein_coding	1	184790724	184974508	-1
ENSG00000121481	RNF2	protein_coding	1	185045526	185102603	1
ENSG00000121486	TRMT1L	protein_coding	1	185118101	185157072	-1
ENSG00000116668	SWT1	protein_coding	1	185157080	185291781	1
ENSG00000116679	IVNS1ABP	protein_coding	1	185296388	185317273	-1

ENSG00000143341	HMCN1	protein_coding	1	185734391	186190949	1
ENSG00000047410	TPR	protein_coding	1	186311652	186375693	-1
ENSG00000157181	ODR4	protein_coding	1	186375838	186421378	1
ENSG00000073756	PTGS2	protein_coding	1	186671791	186680922	-1
ENSG00000116711	PLA2G4A	protein_coding	1	186828949	186988981	1
ENSG00000162670	BRINP3	protein_coding	1	190097658	190478404	-1
ENSG00000253148	RGS21	protein_coding	1	192316992	192367285	1
ENSG00000127074	RGS13	protein_coding	1	192636138	192660311	1
ENSG00000116741	RGS2	protein_coding	1	192809039	192812275	1
ENSG00000116750	UCHL5	protein_coding	1	193012250	193060080	-1
ENSG00000116747	RO60	protein_coding	1	193059454	193091777	1
ENSG00000023572	GLRX2	protein_coding	1	193090866	193106114	-1
ENSG00000134371	CDC73	protein_coding	1	193121983	193254815	1
ENSG00000162630	B3GALT2	protein_coding	1	193178730	193186613	-1
ENSG00000162687	KCNT2	protein_coding	1	196225779	196609225	-1
ENSG00000000971	CFH	protein_coding	1	196652043	196747504	1
ENSG00000244414	CFHR1	protein_coding	1	196819731	196832189	1
ENSG00000134389	CFHR5	protein_coding	1	196977556	197009678	1
ENSG00000066279	ASPM	protein_coding	1	197084121	197146694	-1
ENSG00000177888	ZBTB41	protein_coding	1	197153682	197201293	-1
ENSG00000134376	CRB1	protein_coding	1	197268204	197478455	1
ENSG00000213047	DENND1B	protein_coding	1	197504748	197775696	-1
ENSG00000203724	C1orf53	protein_coding	1	197902630	197907367	1
ENSG00000143355	LHX9	protein_coding	1	197911902	197935478	1
ENSG00000151414	NEK7	protein_coding	1	198156994	198322420	1
ENSG00000081237	PTPRC	protein_coding	1	198638457	198757476	1
ENSG00000116833	NR5A2	protein_coding	1	200027614	200177420	1
ENSG00000162702	ZNF281	protein_coding	1	200404940	200410056	-1
ENSG00000118193	KIF14	protein_coding	1	200551497	200620751	-1
ENSG00000118197	DDX59	protein_coding	1	200623896	200669907	-1
ENSG00000118200	CAMSAP2	protein_coding	1	200738893	200860704	1
ENSG00000163362	INAVA	protein_coding	1	200891048	200915742	1
ENSG00000116852	KIF21B	protein_coding	1	200969390	201023714	-1
ENSG00000081248	CACNA1S	protein_coding	1	201039512	201112451	-1
ENSG00000116857	TMEM9	protein_coding	1	201134772	201171574	-1
ENSG00000163395	IGFN1	protein_coding	1	201190824	201228952	1
ENSG00000159173	TNNI1	protein_coding	1	201403768	201429866	-1
ENSG00000174307	PHLDA3	protein_coding	1	201464278	201469237	-1
ENSG00000159176	CSRP1	protein_coding	1	201483530	201509456	-1
ENSG00000134369	NAV1	protein_coding	1	201539127	201826969	1
ENSG00000198700	IPO9	protein_coding	1	201829149	201884291	1
ENSG00000198892	SHISA4	protein_coding	1	201888680	201892587	1
ENSG00000163431	LMOD1	protein_coding	1	201896456	201946588	-1
ENSG00000134375	TIMM17A	protein_coding	1	201955503	201970664	1
ENSG00000176393	RNPEP	protein_coding	1	201982372	202006147	1
ENSG00000170075	GPR37L1	protein_coding	1	202122886	202133592	1
ENSG00000143862	ARL8A	protein_coding	1	202133404	202144743	-1
ENSG00000133067	LGR6	protein_coding	1	202193799	202319781	1
ENSG00000077152	UBE2T	protein_coding	1	202331657	202341980	-1
ENSG00000077157	PPP1R12B	protein_coding	1	202348699	202592706	1
ENSG00000143858	SYT2	protein_coding	1	202590596	202710454	-1
ENSG00000117139	KDM5B	protein_coding	1	202724495	202808487	-1
ENSG00000183155	RABIF	protein_coding	1	202878282	202889149	-1
ENSG00000117153	KLHL12	protein_coding	1	202891116	202928636	-1
ENSG00000159346	ADIPOR1	protein_coding	1	202940826	202958572	-1
ENSG00000159348	CYB5R1	protein_coding	1	202961873	202967275	-1
ENSG00000163444	TMEM183A	protein_coding	1	203007374	203024848	1
ENSG00000143847	PPFIA4	protein_coding	1	203026491	203078740	1
ENSG00000122180	MYOG	protein_coding	1	203083129	203086012	-1
ENSG00000163485	ADORA1	protein_coding	1	203090654	203167405	1
ENSG00000133048	CHI3L1	protein_coding	1	203178931	203186704	-1
ENSG00000159388	BTG2	protein_coding	1	203305491	203309602	1
ENSG00000122176	FMOD	protein_coding	1	203340628	203351758	-1

ENSG00000188783	PRELP	protein_coding	1	203475806	203491352	1
ENSG00000058668	ATP2B4	protein_coding	1	203626787	203744081	1
ENSG00000257315	ZBED6	protein_coding	1	203795714	203854999	1
ENSG00000182004	SNRPE	protein_coding	1	203861599	203871152	1
ENSG00000143842	SOX13	protein_coding	1	204073115	204127743	1
ENSG00000143845	ETNK2	protein_coding	1	204131062	204152044	-1
ENSG00000174567	GOLT1A	protein_coding	1	204198163	204213988	-1
ENSG00000143850	PLEKHA6	protein_coding	1	204218853	204377665	-1
ENSG00000158615	PPP1R15B	protein_coding	1	204396492	204411887	-1
ENSG00000133056	PIK3C2B	protein_coding	1	204422628	204494805	-1
ENSG00000198625	MDM4	protein_coding	1	204516379	204558120	1
ENSG00000170382	LRRN2	protein_coding	1	204617170	204685738	-1
ENSG00000163531	NFASC	protein_coding	1	204828651	205022822	1
ENSG00000184144	CNTN2	protein_coding	1	205042937	205078289	1
ENSG00000174529	TMEM81	protein_coding	1	205083129	205084460	-1
ENSG00000117222	RBBP5	protein_coding	1	205086142	205122015	-1
ENSG00000133059	DSTYK	protein_coding	1	205142505	205211702	-1
ENSG00000133069	TMCC2	protein_coding	1	205227946	205285632	1
ENSG00000163545	NUAK2	protein_coding	1	205302063	205321745	-1
ENSG00000162873	KLHDC8A	protein_coding	1	205336061	205357090	-1
ENSG00000186007	LEMD1	protein_coding	1	205381378	205455954	-1
ENSG00000281406	BLACAT1	protein_coding	1	205434885	205457091	-1
ENSG00000117266	CDK18	protein_coding	1	205504596	205532793	1
ENSG00000174514	MFSD4A	protein_coding	1	205568885	205602918	1
ENSG00000158711	ELK4	protein_coding	1	205597556	205632011	-1
ENSG00000158715	SLC45A3	protein_coding	1	205657851	205680509	-1
ENSG00000069275	NUCKS1	protein_coding	1	205712822	205750182	-1
ENSG00000117280	RAB29	protein_coding	1	205767986	205775482	-1
ENSG00000133065	SLC41A1	protein_coding	1	205789094	205813748	-1
ENSG00000174502	SLC26A9	protein_coding	1	205913048	205943460	-1
ENSG00000198049	AVPR1B	protein_coding	1	206106936	206117699	-1
ENSG00000196550	FAM72A	protein_coding	1	206186179	206204414	-1
ENSG00000266028	SRGAP2	protein_coding	1	206203346	206464436	1
ENSG00000263528	IKBKE	protein_coding	1	206470476	206496889	1
ENSG00000266094	RASSF5	protein_coding	1	206507531	206589448	1
ENSG00000143486	EIF2D	protein_coding	1	206571292	206612465	-1
ENSG00000143479	DYRK3	protein_coding	1	206635536	206684419	1
ENSG00000162889	MAPKAPK2	protein_coding	1	206684905	206734281	1
ENSG00000123836	PFKFB2	protein_coding	1	207034366	207081024	1
ENSG00000180667	YOD1	protein_coding	1	207043849	207052980	-1
ENSG00000196352	CD55	protein_coding	1	207321532	207386804	1
ENSG00000117335	CD46	protein_coding	1	207752054	207795513	1
ENSG00000174059	CD34	protein_coding	1	207880972	207911402	-1
ENSG00000076356	PLXNA2	protein_coding	1	208022242	208244384	-1
ENSG00000008118	CAMK1G	protein_coding	1	209583714	209613939	1
ENSG00000196878	LAMB3	protein_coding	1	209614870	209652425	-1
ENSG00000123689	G0S2	protein_coding	1	209675412	209676390	1
ENSG00000117594	HSD11B1	protein_coding	1	209686178	209734949	1
ENSG00000162757	C1orf74	protein_coding	1	209779208	209784559	-1
ENSG00000117595	IRF6	protein_coding	1	209785617	209806175	-1
ENSG00000117597	UTP25	protein_coding	1	209827972	209857565	1
ENSG00000143469	SYT14	protein_coding	1	209900923	210171389	1
ENSG00000082497	SERTAD4	protein_coding	1	210232796	210246631	1
ENSG00000054392	HHAT	protein_coding	1	210328252	210676296	1
ENSG00000143473	KCNH1	protein_coding	1	210676823	211134165	-1
ENSG00000117625	RCOR3	protein_coding	1	211258377	211316385	1
ENSG00000082512	TRAF5	protein_coding	1	211326615	211374946	1
ENSG00000198570	RD3	protein_coding	1	211476522	211492162	-1
ENSG00000170385	SLC30A1	protein_coding	1	211571568	211579161	-1
ENSG00000117650	NEK2	protein_coding	1	211658657	211675630	-1
ENSG00000123684	LPGAT1	protein_coding	1	211743457	211830763	-1
ENSG00000143493	INTS7	protein_coding	1	211940399	212035557	-1
ENSG00000143476	DTL	protein_coding	1	212035553	212107400	1

ENSG00000066027	PPP2R5A	protein_coding	1	212285410	212361853	1
ENSG00000065600	PACC1	protein_coding	1	212363928	212414901	-1
ENSG00000117691	NENF	protein_coding	1	212432920	212446379	1
ENSG00000162772	ATF3	protein_coding	1	212565334	212620777	1
ENSG00000123685	BATF3	protein_coding	1	212686417	212699840	-1
ENSG00000117697	NSL1	protein_coding	1	212726153	212791782	-1
ENSG00000203705	TATDN3	protein_coding	1	212791828	212816830	1
ENSG00000185523	SPATA45	protein_coding	1	212830141	212847649	-1
ENSG00000162769	FLVCR1	protein_coding	1	212858275	212899363	1
ENSG00000143494	VASH2	protein_coding	1	212950520	212992037	1
ENSG00000174606	ANGEL2	protein_coding	1	212992182	213015867	-1
ENSG00000136643	RPS6KC1	protein_coding	1	213051233	213274774	1
ENSG00000117707	PROX1	protein_coding	1	213983181	214041510	1
ENSG00000143499	SMYD2	protein_coding	1	214281102	214337131	1
ENSG00000152104	PTPN14	protein_coding	1	214348700	214552449	-1
ENSG00000117724	CENPF	protein_coding	1	214603195	214664571	1
ENSG00000082482	KCNK2	protein_coding	1	215005775	215237090	1
ENSG00000136636	KCTD3	protein_coding	1	215567304	215621807	1
ENSG00000042781	USH2A	protein_coding	1	215622891	216423448	-1
ENSG00000196482	ESRRG	protein_coding	1	216503246	217137755	-1
ENSG00000092978	GPATCH2	protein_coding	1	217426992	217631090	-1
ENSG00000162814	SPATA17	protein_coding	1	217631324	217871696	1
ENSG00000067533	RRP15	protein_coding	1	218285293	218337983	1
ENSG00000092969	TGFB2	protein_coding	1	218345336	218444619	1
ENSG00000143353	LYPLAL1	protein_coding	1	219173869	219212865	1
ENSG00000215817	ZC3H11B	protein_coding	1	219608010	219613145	-1
ENSG00000196660	SLC30A10	protein_coding	1	219685427	219958647	-1
ENSG00000136628	EPRS1	protein_coding	1	219968600	220046530	-1
ENSG00000162813	BPNT1	protein_coding	1	220057482	220090462	-1
ENSG00000067704	IARS2	protein_coding	1	220094132	220148041	1
ENSG00000118873	RAB3GAP2	protein_coding	1	220148293	220272529	-1
ENSG00000116141	MARK1	protein_coding	1	220528136	220664461	1
ENSG00000162817	C1orf115	protein_coding	1	220690363	220699153	1
ENSG00000117791	MTARC2	protein_coding	1	220748225	220784815	1
ENSG00000186205	MTARC1	protein_coding	1	220786913	220819659	1
ENSG00000143507	DUSP10	protein_coding	1	221701424	221742089	-1
ENSG00000143498	TAF1A	protein_coding	1	222557902	222589933	-1
ENSG00000154305	MIA3	protein_coding	1	222618097	222668007	1
ENSG00000186063	AIDA	protein_coding	1	222668013	222713210	-1
ENSG00000162819	BROX	protein_coding	1	222712553	222735196	1
ENSG00000154309	DISP1	protein_coding	1	222815022	223005995	1
ENSG00000187554	TLR5	protein_coding	1	223109404	223143248	-1
ENSG00000143502	SUSD4	protein_coding	1	223220819	223364233	-1
ENSG00000162909	CAPN2	protein_coding	1	223701593	223776018	1
ENSG00000143514	TP53BP2	protein_coding	1	223779893	223845954	-1
ENSG00000143756	FBXO28	protein_coding	1	224114111	224162047	1
ENSG00000143753	DEGS1	protein_coding	1	224175756	224193441	1
ENSG00000143748	NVL	protein_coding	1	224227334	224330189	-1
ENSG00000143771	CNIH4	protein_coding	1	224356858	224379459	1
ENSG00000162923	WDR26	protein_coding	1	224385146	224437033	-1
ENSG00000143786	CNIH3	protein_coding	1	224434660	224740554	1
ENSG00000185842	DNAH14	protein_coding	1	224896262	225399292	1
ENSG00000143815	LBR	protein_coding	1	225401502	225428925	-1
ENSG00000154380	ENAH	protein_coding	1	225486829	225653142	-1
ENSG00000143742	SRP9	protein_coding	1	225777813	225790468	1
ENSG00000143819	EPHX1	protein_coding	1	225810124	225845563	1
ENSG00000196187	TMEM63A	protein_coding	1	225845536	225882380	-1
ENSG00000143811	PYCR2	protein_coding	1	225919877	225924340	-1
ENSG00000143768	LEFTY2	protein_coding	1	225936598	225941383	-1
ENSG00000143751	SDE2	protein_coding	1	225982702	225999343	-1
ENSG00000163041	H3-3A	protein_coding	1	226061851	226072019	1
ENSG00000182827	ACBD3	protein_coding	1	226144679	226186741	-1
ENSG00000183814	LIN9	protein_coding	1	226231149	226309869	-1

ENSG00000143799	PARP1	protein_coding	1	226360210	226408154	-1
ENSG00000203685	STUM	protein_coding	1	226548764	226609230	1
ENSG00000143772	ITPKB	protein_coding	1	226631690	226739323	-1
ENSG00000143801	PSEN2	protein_coding	1	226870184	226927726	1
ENSG00000163050	COQ8A	protein_coding	1	226940286	226987544	1
ENSG00000143776	CDC42BPA	protein_coding	1	226989865	227318492	-1
ENSG00000181450	ZNF678	protein_coding	1	227563543	227677443	1
ENSG00000143740	SNAP47	protein_coding	1	227728200	227781826	1
ENSG00000081692	JMJD4	protein_coding	1	227730425	227735411	-1
ENSG00000143816	WNT9A	protein_coding	1	227918656	227947932	-1
ENSG00000154342	WNT3A	protein_coding	1	228006998	228061271	1
ENSG00000143761	ARF1	protein_coding	1	228082660	228099212	1
ENSG00000143793	C1orf35	protein_coding	1	228100726	228105411	-1
ENSG00000162910	MRPL55	protein_coding	1	228106679	228109312	-1
ENSG00000143774	GUK1	protein_coding	1	228139962	228148984	1
ENSG00000198835	GJC2	protein_coding	1	228149930	228159826	1
ENSG00000181873	IBA57	protein_coding	1	228165804	228182257	1
ENSG00000154358	OBSCN	protein_coding	1	228208044	228378876	1
ENSG00000154370	TRIM11	protein_coding	1	228393673	228406835	-1
ENSG00000162931	TRIM17	protein_coding	1	228407935	228416861	-1
ENSG00000181218	H2AW	protein_coding	1	228434777	228457873	-1
ENSG00000196890	H2BU1	protein_coding	1	228458103	228463104	1
ENSG00000168159	RNF187	protein_coding	1	228487382	228499899	1
ENSG00000116574	RHOU	protein_coding	1	228735479	228746664	1
ENSG00000168118	RAB4A	protein_coding	1	229271062	229305894	1
ENSG00000154429	CCSAP	protein_coding	1	229321011	229343294	-1
ENSG00000143632	ACTA1	protein_coding	1	229430365	229434104	-1
ENSG00000069248	NUP133	protein_coding	1	229440259	229508341	-1
ENSG00000135776	ABCB10	protein_coding	1	229516582	229558707	-1
ENSG00000135801	TAF5L	protein_coding	1	229593121	229626047	-1
ENSG00000135763	URB2	protein_coding	1	229626247	229660200	1
ENSG00000143641	GALNT2	protein_coding	1	230057990	230282122	1
ENSG00000177614	PGBD5	protein_coding	1	230314490	230426332	-1
ENSG00000135775	COG2	protein_coding	1	230642481	230693982	1
ENSG00000135744	AGT	protein_coding	1	230690776	230745576	-1
ENSG00000119280	C1orf198	protein_coding	1	230837119	230869589	-1
ENSG00000143643	TTC13	protein_coding	1	230906243	230978875	-1
ENSG00000173409	ARV1	protein_coding	1	230978981	231000733	1
ENSG00000182118	FAM89A	protein_coding	1	231018958	231040254	-1
ENSG00000119283	TRIM67	protein_coding	1	231162058	231221565	1
ENSG00000143633	C1orf131	protein_coding	1	231223763	231241187	-1
ENSG00000116906	GNPAT	protein_coding	1	231241207	231277973	1
ENSG00000116903	EXOC8	protein_coding	1	231332753	231337852	-1
ENSG00000010072	SPRTN	protein_coding	1	231337104	231355023	1
ENSG00000135766	EGLN1	protein_coding	1	231363751	231422287	-1
ENSG00000116918	TSNAX	protein_coding	1	231528541	231566524	1
ENSG00000162946	DISC1	protein_coding	1	231626790	232041272	1
ENSG00000116991	SIPA1L2	protein_coding	1	232397965	232630571	-1
ENSG00000212916	MAP10	protein_coding	1	232805416	232809929	1
ENSG00000135778	NTPCR	protein_coding	1	232950605	232983882	1
ENSG00000135749	PCNX2	protein_coding	1	232983435	233295725	-1
ENSG00000143674	MAP3K21	protein_coding	1	233327724	233385148	1
ENSG00000135750	KCNK1	protein_coding	1	233614106	233672514	1
ENSG00000183780	SLC35F3	protein_coding	1	233904676	234324511	1
ENSG00000168275	COA6	protein_coding	1	234373456	234385080	1
ENSG00000059588	TARBP1	protein_coding	1	234391313	234479179	-1
ENSG00000168264	IRF2BP2	protein_coding	1	234604269	234610178	-1
ENSG00000173726	TOMM20	protein_coding	1	235109341	235128837	-1
ENSG00000188739	RBM34	protein_coding	1	235131183	235161283	-1
ENSG00000054267	ARID4B	protein_coding	1	235131634	235328219	-1
ENSG00000152904	GGPS1	protein_coding	1	235327350	235344532	1
ENSG00000285053	TBCE	protein_coding	1	235328570	235448952	1
ENSG00000284770	TBCE	protein_coding	1	235367360	235452443	1

ENSG00000162885	B3GALNT2	protein_coding	1	235447190	235504452	-1
ENSG00000168243	GNG4	protein_coding	1	235547685	235650754	-1
ENSG00000143669	LYST	protein_coding	1	235661041	235883640	-1
ENSG00000116962	NID1	protein_coding	1	235975830	236065109	-1
ENSG00000077585	GPR137B	protein_coding	1	236142505	236221865	1
ENSG00000086619	ERO1B	protein_coding	1	236214681	236282019	-1
ENSG00000186197	EDARADD	protein_coding	1	236348257	236502915	1
ENSG00000116977	LGALS8	protein_coding	1	236518000	236552981	1
ENSG00000119285	HEATR1	protein_coding	1	236549005	236604516	-1
ENSG00000077522	ACTN2	protein_coding	1	236664141	236764631	1
ENSG00000116984	MTR	protein_coding	1	236795260	236921278	1
ENSG00000198626	RYR2	protein_coding	1	237042184	237833988	1
ENSG00000133019	CHRM3	protein_coding	1	239386565	239915452	1
ENSG00000155816	FMN2	protein_coding	1	240014348	240475187	1
ENSG00000180875	GREM2	protein_coding	1	240489573	240612155	-1
ENSG00000182901	RGS7	protein_coding	1	240767636	241357374	-1
ENSG00000091483	FH	protein_coding	1	241497511	241519799	-1
ENSG00000117009	KMO	protein_coding	1	241532134	241595642	1
ENSG00000054277	OPN3	protein_coding	1	241590102	241677376	-1
ENSG00000203668	CHML	protein_coding	1	241628851	241640369	-1
ENSG00000174371	EXO1	protein_coding	1	241847967	241895148	1
ENSG00000197769	MAP1LC3C	protein_coding	1	241995490	241999098	-1
ENSG00000180287	PLD5	protein_coding	1	242082986	242524697	-1
ENSG00000143702	CEP170	protein_coding	1	243124428	243255348	-1
ENSG00000054282	SDCCAG8	protein_coding	1	243256034	243500091	1
ENSG00000117020	AKT3	protein_coding	1	243488233	243851079	-1
ENSG00000179456	ZBTB18	protein_coding	1	244048939	244057476	1
ENSG00000035687	ADSS2	protein_coding	1	244408494	244451909	-1
ENSG00000179397	CATSPERE	protein_coding	1	244454377	244641177	1
ENSG00000121644	DESI2	protein_coding	1	244653103	244709033	1
ENSG00000203667	COX20	protein_coding	1	244835616	244845057	1
ENSG00000153187	HNRNPU	protein_coding	1	244840638	244864560	-1
ENSG00000203666	EFCAB2	protein_coding	1	244969682	245127164	1
ENSG00000162849	KIF26B	protein_coding	1	245154985	245709432	1
ENSG00000185420	SMYD3	protein_coding	1	245749342	246507312	-1
ENSG00000162851	TFB2M	protein_coding	1	246540561	246566261	-1
ENSG00000162852	CNST	protein_coding	1	246566444	246668595	1
ENSG00000143653	SCCPDH	protein_coding	1	246724409	246768137	1
ENSG00000153207	AHCTF1	protein_coding	1	246839098	246931948	-1
ENSG00000197472	ZNF695	protein_coding	1	246945547	247008093	-1
ENSG00000277462	ZNF670	protein_coding	1	247034637	247078811	-1
ENSG00000188295	ZNF669	protein_coding	1	247099962	247104372	-1
ENSG00000196418	ZNF124	protein_coding	1	247121975	247172020	-1
ENSG00000162714	ZNF496	protein_coding	1	247297412	247331867	-1
ENSG00000162711	NLRP3	protein_coding	1	247416156	247449108	1
ENSG00000162722	TRIM58	protein_coding	1	247857187	247880138	1
ENSG00000238243	OR2W3	protein_coding	1	247895587	247896531	1
ENSG00000203663	OR2L2	protein_coding	1	248030070	248042305	1
ENSG00000198128	OR2L3	protein_coding	1	248046836	248063407	1
ENSG00000196071	OR2L13	protein_coding	1	248095184	248101103	1
ENSG00000175137	SH3BP5L	protein_coding	1	248810446	248825915	-1
ENSG00000171161	ZNF672	protein_coding	1	248838210	248849517	1
ENSG00000171163	ZNF692	protein_coding	1	248850006	248859144	-1
ENSG00000185220	PGBD2	protein_coding	1	248906196	248919946	1
ENSG00000124786	SLC35B3	protein_coding	6	8411435	8435561	-1
ENSG00000137203	TFAP2A	protein_coding	6	10393186	10419659	-1
ENSG00000111846	GCNT2	protein_coding	6	10492223	10629368	1
ENSG00000137434	C6orf52	protein_coding	6	10671418	10694797	-1
ENSG00000111845	PAK1IP1	protein_coding	6	10694972	10709782	1
ENSG00000111843	TMEM14C	protein_coding	6	10722915	10731129	1
ENSG00000137210	TMEM14B	protein_coding	6	10747759	10852753	1
ENSG00000111837	MAK	protein_coding	6	10762723	10838553	-1
ENSG00000153157	SYCP2L	protein_coding	6	10886831	10979320	1

ENSG00000197977	ELOVL2	protein_coding	6	10980759	11044305	-1
ENSG00000224531	SMIM13	protein_coding	6	11093834	11138733	1
ENSG00000111859	NEDD9	protein_coding	6	11183298	11382348	-1
ENSG00000205269	TMEM170B	protein_coding	6	11537749	11583524	1
ENSG00000111863	ADTRP	protein_coding	6	11712054	11807046	-1
ENSG00000095951	HIVEP1	protein_coding	6	12008762	12164999	1
ENSG00000078401	EDN1	protein_coding	6	12290361	12297194	1
ENSG00000112137	PHACTR1	protein_coding	6	12716312	13290446	1
ENSG00000145979	TBC1D7	protein_coding	6	13266542	13328583	-1
ENSG00000145990	GFOD1	protein_coding	6	13357830	13487662	-1
ENSG00000124523	SIRT5	protein_coding	6	13574227	13615158	1
ENSG00000225921	NOL7	protein_coding	6	13615335	13632739	1
ENSG00000010017	RANBP9	protein_coding	6	13621498	13711835	-1
ENSG00000050393	MCUR1	protein_coding	6	13786557	13814568	-1
ENSG00000180537	RNF182	protein_coding	6	13924446	13980310	1
ENSG00000112149	CD83	protein_coding	6	14117256	14136918	1

Supplementary Table 6. Average values for electrophysiological properties of the iPSC-derived hCS (mean ± SE).

	IV curves	VI curves	Current, Imax (pA)		Voltage, Vt (mV)		Resting potential, RP (mV)		Input resistance, Rin (GΩ)		Threshold current, It (pA)	
	N (cells)	N (cells)	N (cells)	Current, pA	N (cells)	Voltage, mV	N (cells)	Voltage, mV	N (cells)	Resistance, GΩ	N (cells)	Current, pA
CTRL	29	14	29	-137.25 ± 22.26	29	-17.96 ± 2.29	14	-65.27 ± 1.21	12	0.27 ± 0.03	18	46.68 ± 9.96
CTRL + Li	29	18	29	-125.82 ± 18.58	29	-15.08 ± 1.92	22	-61.28 ± 1.07	19	0.26 ± 0.02	9	44.93 ± 5.90
Li-N	8	6	20	-148.99 ± 66.60	20	-10.30 ± 2.81	3	-57.85 ± 2.38	9	0.38 ± 0.05	10	42.01 ± 9.89
Li-N + Li	10	2	19	-245.83 ± 96.29	19	-22.14 ± 4.86	6	-60.96 ± 2.51	11	0.31 ± 0.02	10	35.53 ± 4.51
Li-R	20	10	8	-125.46 ± 26.13	8	-13.57 ± 2.31	8	-66.58 ± 2.06	4	0.28 ± 0.03	6	48.76 ± 8.85
Li-R + Li	19	10	10	-334.80 ± 99.03	10	-15.49 ± 2.10	11	-65.40 ± 2.09	1	0.35 ± 0.06	2	34.99 ± 10.84
BD	28	16	28	-132.18 ± 26.00	28	-12.64 ± 1.83	11	-65.02 ± 1.84	15	0.26 ± 0.03	16	46.23 ± 6.51
BD + Li	29	12	29	-304.12 ± 72.26	29	-17.78 ± 2.20	17	-63.00 ± 1.77	11	0.35 ± 0.06	12	35.08 ± 8.96

Supplementary Table 7. sEPSC distribution peak and mean amplitudes and frequencies.

			sEPSC distribution (pA)		
	N. (donors)	N. (cells)	Amplitude peak	Frequency peak	Amplitude mean ± SE
CTRL	10	47	4.91 ± 0.02	3.04 ± 0.03	5,68 ± 1,42
CTRL + Li	10	42	5.7 ± 0.02	3.48 ± 0.05	5,55 ± 1,2
Li-N	5	14	5.01 ± 0.04	2.64 ± 0.04	5,33 ± 0,54
Li-N + Li	4	13	6.51 ± 0.06	4.27 ± 0.06	6,48 ± 2,01
Li-R	5	23	7.23 ± 0.05	2.3 ± 0.03	6,6 ± 2,1
Li-R + Li	5	16	4.61 ± 0.11	3.995 ± 0.11	6,92 ± 2,15
BD	10	37	6.28 ± 0.06	2.42 ± 0.03	5,98 ± 1,59
BD + Li	9	29	5.58 ± 0.1	4.13 ± 0.06	6,72 ± 1,97

Supplementary Table 8. Biological functions of the 3 diagnosis-associated DEGs.

ensembl_gene_id	gene_name	gene_biotype	chromosome	go_id	Biological functions
ENSG00000120215	MLANA	protein_coding	9	GO:0042470	melanosome
ENSG00000120215	MLANA	protein_coding	9	GO:0005789	endoplasmic reticulum membrane
ENSG00000120215	MLANA	protein_coding	9	GO:0005794	Golgi apparatus
ENSG00000120215	MLANA	protein_coding	9	GO:0016021	integral component of membrane
ENSG00000120215	MLANA	protein_coding	9	GO:0005887	integral component of plasma membrane
ENSG00000120215	MLANA	protein_coding	9	GO:0005783	endoplasmic reticulum
ENSG00000120215	MLANA	protein_coding	9		
ENSG00000120215	MLANA	protein_coding	9	GO:0016020	membrane
ENSG00000120215	MLANA	protein_coding	9	GO:0005802	trans-Golgi network
ENSG00000120215	MLANA	protein_coding	9	GO:0005515	protein binding
ENSG00000196604	POTEF	protein_coding	2	GO:0005615	extracellular space
ENSG00000196604	POTEF	protein_coding	2	GO:0001895	retina homeostasis
ENSG00000196604	POTEF	protein_coding	2	GO:0072562	blood microparticle
ENSG00000196604	POTEF	protein_coding	2	GO:0005515	protein binding
ENSG00000196604	POTEF	protein_coding	2	GO:0070062	extracellular exosome
ENSG00000196604	POTEF	protein_coding	2	GO:0005737	cytoplasm
ENSG00000196604	POTEF	protein_coding	2	GO:0005938	cell cortex
ENSG00000129204	USP6	protein_coding	17	GO:0008234	cysteine-type peptidase activity
ENSG00000129204	USP6	protein_coding	17	GO:0005737	cytoplasm
ENSG00000129204	USP6	protein_coding	17	GO:0004197	cysteine-type endopeptidase activity
ENSG00000129204	USP6	protein_coding	17	GO:0006511	ubiquitin-dependent protein catabolic process
ENSG00000129204	USP6	protein_coding	17	GO:0004843	cysteine-type deubiquitinase activity
ENSG00000129204	USP6	protein_coding	17	GO:0055037	recycling endosome
ENSG00000129204	USP6	protein_coding	17	GO:0008233	peptidase activity
ENSG00000129204	USP6	protein_coding	17	GO:0005768	endosome
ENSG00000129204	USP6	protein_coding	17	GO:0036211	protein modification process
ENSG00000129204	USP6	protein_coding	17	GO:0016579	protein deubiquitination
ENSG00000129204	USP6	protein_coding	17	GO:0006508	proteolysis
ENSG00000129204	USP6	protein_coding	17	GO:0016787	hydrolase activity
ENSG00000129204	USP6	protein_coding	17		
ENSG00000129204	USP6	protein_coding	17	GO:0060627	regulation of vesicle-mediated transport
ENSG00000129204	USP6	protein_coding	17	GO:0003676	nucleic acid binding
ENSG00000129204	USP6	protein_coding	17	GO:0005515	protein binding

ENSG00000129204	USP6	protein_coding	17	GO:0005886	plasma membrane
ENSG00000129204	USP6	protein_coding	17	GO:0016020	membrane
ENSG00000129204	USP6	protein_coding	17	GO:0005096	GTPase activator activity
ENSG00000129204	USP6	protein_coding	17	GO:0005764	lysosome
ENSG00000129204	USP6	protein_coding	17	GO:0005516	calmodulin binding
ENSG00000129204	USP6	protein_coding	17	GO:0090630	activation of GTPase activity

Supplementary Table 9. DESeq2 DEGs associated to Li treatment independent of diagnosis.

ensembl_gene_id	external_gene_name	baseMean	log2FoldChange	pvalue	padj	gene_biotype	chromosome
ENSG00000131378	RFTN1	175 192 834 347 388	-0.555043555159832	3,95E+06	0.00053380011	protein_coding	3
ENSG00000103260	METRN	478 751 172 355 966	-0.557737726229716	7,60E+07	0.00441749474	protein_coding	16
ENSG00000177791	MYOZ1	155 962 344 305 493	-242 603 442 907 202	9,81E+07	0.00441749474	protein_coding	10
ENSG00000072954	TMEM38A	706 333 416 538 511	-0.474777718807413	1,90E+08	0.00630426161	protein_coding	19
ENSG00000036549	ZZZ3	579 791 648 389 521	0.165324943409407	2,33E+07	0.00630426161	protein_coding	1
ENSG00000104267	CA2	269 679 988 129 886	-0.841185912304932	3,19E+07	0.00719375853	protein_coding	8
ENSG00000172403	SYNPO2	147 657 553 550 535	-166 484 999 641 287	4,02E+08	0.00775716591	protein_coding	4
ENSG00000196972	SMIM10L2B	516 287 560 716 497	-0.563250422630353	4,83E+08	0.00816449965	protein_coding	X
ENSG00000197852	INKA2	181 009 515 517 406	-0.477644857224827	1,02E+09	0.01527166994	protein_coding	1
ENSG00000009830	POMT2	107 435 957 419 104	-0.292316956180705	1,26E+08	0.01597598978	protein_coding	14
ENSG00000186377	CYP4X1	256 464 960 504 147	-0.69696012077972	1,38E+09	0.01597598978	protein_coding	1
ENSG00000149809	TM7SF2	857 345 110 080 663	-0.423507428959903	1,47E+09	0.01597598978	protein_coding	11
ENSG00000027847	B4GALT7	44 041 085 752 189	-0.330501549995926	1,54E+09	0.01597598978	protein_coding	5
ENSG00000113494	PRLR	907 019 380 462 046	-0.858969292413332	1,71E+09	0.01649455511	protein_coding	5
ENSG00000108641	B9D1	918 789 657 817 158	-0.378649100279797	2,07E+08	0.01703579265	protein_coding	17
ENSG00000172426	RSPH9	12 843 651 358 131	-100 437 437 458 145	2,10E+09	0.01703579265	protein_coding	6
ENSG00000187094	CCK	101 870 095 992 394	-153 331 847 980 226	2,14E+08	0.01703579265	protein_coding	3
ENSG00000110195	FOLR1	116 361 072 925 286	-105 013 620 391 489	2,48E+09	0.01859071691	protein_coding	11
ENSG00000067334	DNTTIP2	440 009 072 062 087	0.208632244864113	2,81E+08	0.01910177715	protein_coding	1
ENSG00000136918	WDR38	629 495 811 555 246	-202 824 617 288 951	2,83E+09	0.01910177715	protein_coding	9
ENSG00000103249	CLCN7	76 751 496 632 884	-0.445155759843033	3,57E+09	0.02295595717	protein_coding	16
ENSG00000152465	NMT2	195 139 046 158 907	0.226210978006453	5,15E+09	0.02987193822	protein_coding	10
ENSG00000167280	ENGASE	42 031 596 923 198	-0.405537737182941	5,53E+09	0.02987193822	protein_coding	17
ENSG00000169116	PARM1	290 091 553 187 506	-0.546167994423913	5,67E+08	0.02987193822	protein_coding	4
ENSG00000066422	ZBTB11	292 854 477 589 332	0.191828588461687	5,77E+09	0.02987193822	protein_coding	3
ENSG00000127329	PTPRB	490 454 554 417 167	-200 165 982 476 825	5,96E+09	0.02987193822	protein_coding	12
ENSG00000163817	SLC6A20	185 977 348 911 689	-171 038 162 438 627	5,97E+09	0.02987193822	protein_coding	3
ENSG00000196504	PRPF40A	780 107 807 311 314	0.191795943250063	6,41E+09	0.03091240643	protein_coding	2
ENSG00000123360	PDE1B	172 112 806 835 114	-0.769134057629573	6,87E+09	0.03202344270	protein_coding	12
ENSG00000125817	CENPB	249 899 571 263 747	-0.282411865097923	8,34E+09	0.03740929273	protein_coding	20
ENSG00000011105	TSPAN9	172 596 040 736 509	-0.409505557463217	8,58E+09	0.03740929273	protein_coding	12
ENSG00000159792	PSKH1	10 463 431 859 635	-0.318313248070991	9,10E+09	0.03842522389	protein_coding	16
ENSG00000176102	CSTF3	379 563 014 332 337	0.206943128654847	1002051274	0.03992825593	protein_coding	11

ENSG00000161921	CXCL16	264 464 074 939 577	-0.547562968901328	1004781808	0.03992825593	protein_coding	17
ENSG00000083520	DIS3	472 313 955 116 344	0.20920554987262	0104130059	0.04019717820	protein_coding	13
ENSG00000136546	SCN7A	162 198 821 766 013	-136 911 729 892 876	1097722524	0.04119813619	protein_coding	2
ENSG00000115665	SLC5A7	117 779 074 179 562	-200 778 049 971 605	1279058717	0.04572325462	protein_coding	2
ENSG00000165732	DDX21	806 637 297 443 062	0.229443738384323	1285977111	0.04572325462	protein_coding	10
ENSG00000004838	ZMYND10	561 791 570 919 461	-0.690624054526208	1366714256	0.04644541931	protein_coding	3
ENSG00000178605	GTPBP6	348 970 962 824 335	-0.419685563727088	1375040169	0.04644541931	protein_coding	X
ENSG00000187783	TMEM72	129 448 945 206 322	-21 668 596 447 478	1447845364	0.04771180176	protein_coding	10
ENSG00000185909	KLHDC8B	123 762 049 968 987	-0.427559626101462	1537092136	0.04848717992	protein_coding	3
ENSG00000105048	TNNT1	401 827 937 735 273	-0.783106699685736	1653713860	0.04848717992	protein_coding	19
ENSG00000171551	ECEL1	421 925 318 214 486	-109 638 319 810 681	01676244264	0.04848717992	protein_coding	2
ENSG00000008283	CYB561	13 282 939 450 054	-0.452664326127894	1685245175	0.04848717992	protein_coding	17
ENSG00000174206	C12orf66	633 585 226 030 145	0.32488874979195	1685368496	0.04848717992	protein_coding	12
ENSG00000101892	ATP1B4	146 814 371 871 323	-734 756 448 677 438	1713152185	0.04848717992	protein_coding	X
ENSG00000164896	FASTK	54 003 970 921 767	-0.418893098514553	1722585031	0.04848717992	protein_coding	7
ENSG00000197619	ZNF615	16 667 451 720 834	0.254890273493428	1789710631	0.04934853131	protein_coding	19
ENSG00000013563	DNASE1L1	39 545 695 625 342	-0.622525268081986	2007084821	0.05423544604	protein_coding	X
ENSG00000129654	FOXJ1	23 370 630 210 202	-102 583 825 645 239	2056500607	0.05448113668	protein_coding	17
ENSG00000110002	VWA5A	176 670 596 890 687	-0.334107623858099	2130060197	0.05483524236	protein_coding	11
ENSG00000154319	FAM167A	722 522 257 847 183	-0.389712476742377	2151038298	0.05483524236	protein_coding	8
ENSG00000174417	TRHR	391 024 054 457 546	-144 709 257 584 596	2334056324	0.05839895369	protein_coding	8
ENSG00000167635	ZNF146	752 365 096 344 333	0.207170095939281	2608335098	0.06407493730	protein_coding	19
ENSG00000033800	PIAS1	579 586 798 944 208	0.135806953895471	2797933038	0.06723620217	protein_coding	15
ENSG00000139874	SSTR1	215 874 095 655 586	-0.699572282144335	02850982569	0.06723620217	protein_coding	14
ENSG00000011426	ANLN	127 227 196 474 462	0.499485612442429	2886314651	0.06723620217	protein_coding	7
ENSG00000108830	RND2	293 543 033 706 698	-0.437593739439257	02937522747	0.06726927092	protein_coding	17
ENSG00000165156	ZHX1	614 666 007 802 392	0.161840382124044	3458157593	0.07584010991	protein_coding	8
ENSG00000224383	PRR29	234 552 459 560 346	-0.877450746904662	3474446389	0.07584010991	protein_coding	17
ENSG00000182481	KPNA2	927 500 780 102 995	0.221559072951534	3637641989	0.07584010991	protein_coding	17
ENSG00000130935	NOL11	419 755 937 933 237	0.19113669712898	3643650423	0.07584010991	protein_coding	17
ENSG00000134042	MRO	931 736 727 094 177	-0.472611230153502	3646847063	0.07584010991	protein_coding	18
ENSG00000167778	SPRYD3	438 805 681 726 775	-0.226090289636429	3701154402	0.07584010991	protein_coding	12
ENSG00000185813	PCYT2	741 750 375 732 286	-0.273024043028528	3741371599	0.07584010991	protein_coding	17
ENSG00000277893	SRD5A2	475 739 250 870 509	-222 135 067 588 559	3760852167	0.07584010991	protein_coding	2
ENSG00000128602	SMO	107 388 226 521 979	-0.303402069141565	3977873817	0.07833798023	protein_coding	7
ENSG00000121871	SLITRK3	131 396 628 202 438	-0.48046860382266	4078132324	0.07833798023	protein_coding	3
ENSG00000221968	FADS3	320 661 789 409 149	-0.42947274776868	4106701601	0.07833798023	protein_coding	11

ENSG00000138639	ARHGAP24	265 889 980 560 231	-0.969205541514586	4116643176	0.0783379802	protein_coding	4
ENSG00000102452	NALCN	762 075 530 525 032	-0.453673331171091	4311881228	0.0809136489	protein_coding	13
ENSG00000205593	DENND6B	569 632 750 189 857	-0.437828507310755	4412862155	0.0816742199	protein_coding	22
ENSG00000118307	CFAP94	470 671 967 368 374	-0.438434610623538	4545859844	0.0828445003	protein_coding	12
ENSG00000196586	MYO6	470 793 495 556 247	0.241351564340256	46225165511	0.0828445003	protein_coding	6
ENSG00000159423	ALDH4A1	585 070 733 607 868	-0.43422144896364	4693164093	0.0828445003	protein_coding	1
ENSG00000119333	DYNC2I2	422 840 612 222 333	-0.363330076571515	4721357804	0.0828445003	protein_coding	9
ENSG00000131747	TOP2A	106 534 852 480 994	0.350456386070379	4800399450	0.0831515345	protein_coding	17
ENSG00000176383	B3GNT4	18 419 743 203 715	-0.57278881140249	5014460421	0.0852656825	protein_coding	12
ENSG00000088727	KIF9	517 839 931 879 325	-0.506431852500873	5048667459	0.0852656825	protein_coding	3
ENSG00000078596	ITM2A	620 753 255 178 548	-0.927457089221854	5298199597	0.0879071665	protein_coding	X
ENSG00000162607	USP1	55 148 432 136 513	0.225420718601539	5335199213	0.0879071665	protein_coding	1
ENSG00000007516	BAIAP3	339 096 585 245 266	-0.679522262491014	5430965585	0.0884069590	protein_coding	16
ENSG00000237172	B3GNT9	370 660 232 358 058	-0.506546356366944	5534072999	0.0890129289	protein_coding	16
ENSG00000025770	NCAPH2	125 876 233 936 673	-0.220942446100909	6030915622	0.0958631776	protein_coding	22
ENSG00000215375	MYL5	279 914 002 804 856	-0.636609219533869	6106638684	0.0959381340	protein_coding	4
ENSG00000131899	LLGL1	358 661 950 419 902	-0.258186387733719	6467659258	0.0999511237	protein_coding	17
ENSG00000270885	RASL10B	125 121 814 981 415	-0.284875359663121	6511495099	0.0999511237	protein_coding	17
ENSG00000120647	CCDC77	721 488 515 991 683	0.265043229372791	6584005633	0.0999511237	protein_coding	12

Supplementary Table 10. Limma DEGs associated to Li treatment independent of diagnosis.

ensembl_gene_id	external_gene_name	logFC	AveExpr	P.Value	adj.P.Val	gene_biotype	chromosome
ENSG00000136546	SCN7A	-19 193 116 578 872	-0.0686384617416382	2,64E+07	0.00387211215078049	protein_coding	2
ENSG00000172426	RSPH9	-0.948280658087842	0.589128250863159	2,57E+07	0.0187894869450047	protein_coding	6
ENSG00000177791	MYOZ1	-256 731 391 268 648	-11 521 022 158 116	8,55E+08	0.0287910849891555	protein_coding	10
ENSG00000170965	PLAC1	-24 792 658 633 818	-440 840 504 545 152	9,48E+08	0.0287910849891555	protein_coding	X
ENSG00000131378	RFTN1	-0.630963465715523	486 641 375 263 335	9,83E+08	0.0287910849891555	protein_coding	3
ENSG00000185274	GALNT17	-0.515180695582278	415 771 956 946 294	1,41E+09	0.0300849052630678	protein_coding	7
ENSG00000103260	METRN	-0.574961637885102	299 399 840 225 313	1,58E+08	0.0300849052630678	protein_coding	16
ENSG00000140285	FGF7	-154 160 084 759 888	-0.15314019024482	1,64E+09	0.0300849052630678	protein_coding	15
ENSG00000120729	MYOT	-313 723 876 164 916	-188 997 974 652 946	2,26E+09	0.0367101699384282	protein_coding	5
ENSG00000172403	SYNPO2	-198 408 812 622 453	-0.127251804354953	3,22E+09	0.0432443071451568	protein_coding	4
ENSG00000011105	TSPAN9	-0.453302918529912	492 125 113 119 313	3,25E+09	0.0432443071451568	protein_coding	12
ENSG00000161395	PGAP3	-0.36419308830981	29 829 915 102 989	4,02E+09	0.0471786374460244	protein_coding	17
ENSG00000126778	SIX1	-254 045 024 414 143	-281 566 052 280 724	4,38E+09	0.0471786374460244	protein_coding	14
ENSG00000180772	AGTR2	-268 725 211 281 566	-393 507 583 258 528	4,51E+09	0.0471786374460244	protein_coding	X
ENSG00000106003	LFNG	-0.603068503886395	230 112 921 840 937	5,56E+09	0.0521090163755627	protein_coding	7
ENSG00000148341	SH3GLB2	-0.295366546158792	471 945 165 011 894	5,69E+09	0.0521090163755627	protein_coding	9
ENSG00000078596	ITM2A	-112 240 164 144 308	204 447 038 200 547	6,83E+09	0.0529864323856416	protein_coding	X
ENSG00000106333	PCOLCE	-0.911414462865161	11 525 748 139 363	6,91E+09	0.0529864323856416	protein_coding	7
ENSG00000123360	PDE1B	-0.702724588305611	149 576 945 746 264	7,50E+09	0.0529864323856416	protein_coding	12
ENSG00000197594	ENPP1	-173 871 741 560 702	-14 793 514 284 152	7,62E+09	0.0529864323856416	protein_coding	6
ENSG00000006128	TAC1	-123 453 720 284 489	180 211 096 664 434	7,87E+09	0.0529864323856416	protein_coding	7
ENSG00000172830	SSH3	-0.67654100415467	1 039 469 041 569	7,96E+09	0.0529864323856416	protein_coding	11
ENSG00000198542	ITGBL1	-176 369 918 290 496	-379 923 124 889 736	8,68E+09	0.0550608050165492	protein_coding	13
ENSG00000196972	SMIM10L2B	-0.586686696052038	309 968 927 657 468	9,02E+09	0.0550608050165492	protein_coding	X
ENSG00000139874	SSTR1	-0.894024828620342	151 322 013 836 659	9,82E+09	0.0575244793636911	protein_coding	14
ENSG00000136918	WDR38	-181 187 303 124 367	-232 811 318 976 396	0.00010543	0.0593957585814828	protein_coding	9
ENSG00000134516	DOCK2	-205 717 533 716 147	-453 770 414 587 903	0.00011796	0.0615355281515627	protein_coding	5
ENSG00000153012	LGI2	-0.910044659911718	35 184 400 753 378	0.00011831	0.0615355281515627	protein_coding	4
ENSG00000106018	VIPR2	-0.969790320260204	181 097 535 432 488	0.00012354	0.0615355281515627	protein_coding	7
ENSG00000196666	FAM180B	-208 860 355 481 051	-391 885 222 378 908	0.00012603	0.0615355281515627	protein_coding	11
ENSG00000184388	PABPC1L2B	-0.973111026405018	0.127283376413454	0.00013884	0.0644507035579898	protein_coding	X
ENSG00000105048	TNNT1	-0.938424491410847	251 092 621 929 549	0.00014080	0.0644507035579898	protein_coding	19
ENSG00000184254	ALDH1A3	-177 726 339 222 715	-316 008 604 235 663	0.00017159	0.0748268239003454	protein_coding	15

ENSG0000011293(	C7	-156 180 692 682 313	-197 246 428 346 232	0.00017618	0.0748268239003454	protein_coding	5
ENSG0000016362:	NKX6-1	-190 956 361 279 494	-411 875 112 851 978	0.00018350	0.0748268239003454	protein_coding	4
ENSG0000013767:	TRPC6	-183 082 111 157 521	-157 814 635 619 727	0.00018391	0.0748268239003454	protein_coding	11
ENSG0000021537!	MYL5	-0.694683262896901	213 934 046 048 849	0.00019287	0.0758019522446452	protein_coding	4
ENSG0000012900!	ISLR	-0.910164331591023	160 241 445 759 256	0.00019665	0.0758019522446452	protein_coding	15
ENSG0000016487!	CA3	-116 276 412 728 465	-13 674 563 058 688	0.00021937	0.0807126790889217	protein_coding	8
ENSG0000010680!	OGN	-140 783 877 355 265	0.679925271969245	0.00022170	0.0807126790889217	protein_coding	9
ENSG0000010324!	CLCN7	-0.430936415706777	381 379 374 212 332	0.00023103	0.0807126790889217	protein_coding	16
ENSG0000019785:	INKA2	-0.536147061993593	498 951 668 298 113	0.00023144	0.0807126790889217	protein_coding	1
ENSG0000014997:	CNTN5	-10 128 668 387 008	0.890153343153212	0.00024393	0.0815515616203357	protein_coding	11
ENSG0000013863!	ARHGAP24	-105 168 730 353 882	131 482 226 148 008	0.00024498	0.0815515616203357	protein_coding	4
ENSG0000017170(	RGS19	-0.651796298114682	0.351475571268373	0.00026408	0.0831367880183973	protein_coding	20
ENSG0000014553(	ADAMTS16	-0.923588314069969	0.854158782923852	0.00026919	0.0831367880183973	protein_coding	5
ENSG0000014457!	CTDSP1	-0.350152954432486	349 135 977 673 045	0.00026928	0.0831367880183973	protein_coding	2
ENSG0000015106:	CACNA2D4	-249 714 790 846	-497 525 645 600 014	0.00027244	0.0831367880183973	protein_coding	12
ENSG0000015353:	ADPRHL1	-18 172 034 514 103	-0.170329652354852	0.00028784	0.0860412079069843	protein_coding	13
ENSG0000024305(	EIF4EBP3	-136 940 243 878 432	-214 291 711 734 009	0.00031080	0.0910465109169024	protein_coding	5
ENSG0000019744	OGDHL	-0.583166536997694	295 900 016 321 581	0.00031786	0.0912907753661035	protein_coding	10
ENSG0000011068(	CALCA	-18 701 765 301 674	-387 916 856 438 467	0.00033084	0.0928769189029955	protein_coding	11
ENSG0000017860!	GTPBP6	-0.46535810665651	262 525 843 271 969	0.00034366	0.0928769189029955	protein_coding	X
ENSG0000007480:	SLC12A1	-129 762 539 098 225	-1 995 900 460 749	0.00034412	0.0928769189029955	protein_coding	15
ENSG0000012892!	IVD	-0.278544938687537	52 312 389 922 567	0.00034875	0.0928769189029955	protein_coding	15
ENSG0000010352!	SYT17	-0.627320419536548	250 261 790 724 108	0.00036971	0.0945420709588773	protein_coding	16
ENSG0000018689	C1QL4	-111 383 490 045 622	-0.172459804798097	0.00037000	0.0945420709588773	protein_coding	12
ENSG0000012360!	NMI	-110 001 308 254 406	-0.514656576430908	0.00037955	0.0945420709588773	protein_coding	2
ENSG0000012860:	SMO	-0.289554957029797	419 616 190 470 849	0.00038082	0.0945420709588773	protein_coding	7
ENSG0000011569	STK25	-0.263703378138167	575 744 769 154 945	0.00039620	0.0954820998309771	protein_coding	2
ENSG0000011566!	SLC5A7	-227 089 890 608 776	-246 985 871 736 078	0.00039813	0.0954820998309771	protein_coding	2
ENSG0000017441	TRHR	-154 415 496 122 971	-161 036 533 315 012	0.00041785	0.0954820998309771	protein_coding	8
ENSG0000006465!	EYA2	-0.87176645685881	0.277518742980199	0.00042587	0.0954820998309771	protein_coding	20
ENSG0000023947	KLHL41	-234 535 315 205 956	119 995 198 520 988	0.00042668	0.0954820998309771	protein_coding	2
ENSG0000010883(	RND2	-0.47640180836585	543 726 306 433 616	0.00042969	0.0954820998309771	protein_coding	17
ENSG0000017420(	C12orf66	0.317331356083567	356 400 043 417 408	0.00043024	0.0954820998309771	protein_coding	12
ENSG0000010315	NECAB2	-0.553685628892386	327 820 611 575 989	0.00044899	0.0981554797356248	protein_coding	16

Supplementary Table 11. Gene ontology (GO) pathway enrichment analysis of Li associated DEGs independent of diagnosis.

ID	Description	GeneRatio	BgRatio	pvalue	p.adjust	geneID	Count
GO:0055078	sodium ion homeostasis	5/116	54/18862	2,01E+09	0.0404565529546236	TAC1/SLC12A1/C7/SCN7A/AGTR2	5
GO:0090190	positive regulation of branching involved in ureteric bud morphogenesis	3/116	17/18862	0.000144722796681	0.0551733175662849	SIX1/SMO/AGTR2	3
GO:0001657	ureteric bud development	5/116	86/18862	0.000188914553529	0.0551733175662849	SIX1/SMO/FOXJ1/ADAMTS16/AGTR2	5
GO:0072163	mesonephric epithelium development	5/116	87/18862	0.000199458688885	0.0551733175662849	SIX1/SMO/FOXJ1/ADAMTS16/AGTR2	5
GO:0072164	mesonephric tubule development	5/116	87/18862	0.000199458688885	0.0551733175662849	SIX1/SMO/FOXJ1/ADAMTS16/AGTR2	5
GO:0060993	kidney morphogenesis	5/116	88/18862	0.000210445632930	0.0551733175662849	SIX1/SMO/FOXJ1/ADAMTS16/AGTR2	5
GO:0060688	regulation of morphogenesis of a branching structure	4/116	48/18862	0.000214450029277	0.0551733175662849	SIX1/SMO/FGF7/AGTR2	4
GO:0090189	regulation of branching involved in ureteric bud morphogenesis	3/116	20/18862	0.000239379927085	0.0551733175662849	SIX1/SMO/AGTR2	3
GO:0001823	mesonephros development	5/116	91/18862	0.000246187336686	0.0551733175662849	SIX1/SMO/FOXJ1/ADAMTS16/AGTR2	5
GO:0044458	motile cilium assembly	3/116	23/18862	0.000366911184333	0.0628661026334277	ZMYND10/FOXJ1/RSPH9	3
GO:0055002	striated muscle cell development	5/116	103/18862	0.000437118714506	0.0628661026334277	TNNT1/SIX1/SMO/MYOZ1/KLHL41	5
GO:1905330	regulation of morphogenesis of an epithelium	4/116	61/18862	0.000540885853740	0.0641745157055443	SIX1/SMO/FGF7/AGTR2	4
GO:0090183	regulation of kidney development	3/116	30/18862	0.000815190034169	0.0745271698571588	SIX1/SMO/AGTR2	3
GO:0008306	associative learning	4/116	68/18862	0.000816672707721	0.0745271698571588	TAC1/PIAS1/PDE1B/CCK	4
GO:0048147	negative regulation of fibroblast proliferation	3/116	31/18862	0.000898504791117	0.0745271698571588	B4GALT7/AGTR2/C1QL4	3
GO:0048741	skeletal muscle fiber development	3/116	31/18862	0.000898504791117	0.0745271698571588	SIX1/SMO/KLHL41	3

Supplementary Table 12. DESeq2 DEGs associated to Li treatment in CTRL hCS.

ensembl_gene_id	external_gene_name	log2FoldChange	pvalue	padj	gene_biotype	chromosome
ENSG0000016591	RAPSN	-214 702 950 048 087	1,59E+02	2,28E+06	protein_coding	11
ENSG0000010326	METRN	-0.731690315758422	3,03E+07	0.00217840541200	protein_coding	16
ENSG0000008751	TFAP2C	0.576066268253775	1,75E+08	0.00813620971451	protein_coding	20
ENSG0000017100	HS6ST2	-0.411491841628401	2,69E+08	0.00813620971451	protein_coding	X
ENSG0000013137	RFTN1	-0.712550415422766	3,51E+08	0.00813620971451	protein_coding	3
ENSG0000017779	MYOZ1	-277 220 522 669 059	3,78E+08	0.00813620971451	protein_coding	10
ENSG0000019697	SMIM10L2B	-0.666158538488588	4,42E+08	0.00813620971451	protein_coding	X
ENSG0000012543	HS3ST3B1	-0.74973644649467	4,53E+08	0.00813620971451	protein_coding	17
ENSG0000013654	SCN7A	-179 509 710 897 342	5,21E+08	0.00830946316163	protein_coding	2
ENSG0000010245	NALCN	-0.733625754993478	7,98E+08	0.01076373453355	protein_coding	13
ENSG0000012258	NXPH1	-0.927892655364668	8,24E+08	0.01076373453355	protein_coding	7
ENSG0000017616	FOXG1	0.404791519515822	1,52E+09	0.01814785672447	protein_coding	14
ENSG0000013404	MRO	-0.611722407832637	1,77E+08	0.01955366673264	protein_coding	18
ENSG0000015382	KCNJ16	-0.610700108464302	2,67E+09	0.02642975047053	protein_coding	17
ENSG0000020332	ZNF525	0.379056606346496	2,76E+09	0.02642975047053	protein_coding	19
ENSG0000017894	SMIM10L2A	-0.412549493788672	2,96E+09	0.02657954237532	protein_coding	X
ENSG0000019681	CHRNG	-126 249 027 592 034	3,24E+09	0.02722995271986	protein_coding	2
ENSG0000011698	HPCAL4	-0.436911509923494	3,41E+09	0.02722995271986	protein_coding	1
ENSG0000010324	CLCN7	-0.522262346388195	4,07E+09	0.03078054502107	protein_coding	16
ENSG0000017743	NAP1L5	-0.336669469269114	4,47E+09	0.03212377217140	protein_coding	4
ENSG0000018267	TTC3	0.34044489066442	6,34E+09	0.03971195519352	protein_coding	21
ENSG0000015532	GRAMD2B	-0.999105511733691	6,45E+09	0.03971195519352	protein_coding	5
ENSG0000014552	CDH18	-0.887314647989896	6,46E+09	0.03971195519352	protein_coding	5
ENSG0000006430	CDON	0.447895846939786	6,76E+09	0.03971195519352	protein_coding	11
ENSG0000013174	TOP2A	0.320489082143432	7,01E+08	0.03971195519352	protein_coding	17
ENSG0000015418	ANGPT1	-0.70440194351079	7,36E+09	0.03971195519352	protein_coding	8
ENSG0000015301	LGI2	-0.847911246332731	7,47E+09	0.03971195519352	protein_coding	4
ENSG0000010868	ASIC2	-102 571 309 873 294	7,76E+08	0.03980158529954	protein_coding	17
ENSG0000018590	KLHDC8B	-0.414893529863307	8,25E+09	0.04084666207975	protein_coding	3
ENSG0000015276	DYNLT5	-157 980 589 343 649	8,59E+09	0.04110259453452	protein_coding	1
ENSG0000016579	NDRG2	-0.490872099248556	9,04E+09	0.04169615754514	protein_coding	14
ENSG0000014594	MYLK4	-135 109 570 778 256	9,29E+09	0.04169615754514	protein_coding	6
ENSG0000015464	CHODL	-0.714118833117481	9,93E+09	0.04241825226597	protein_coding	21

ENSG0000018581	PCYT2	-0.430416730704238	0.000101744	0.04241825226597	protein_coding	17
ENSG0000013264	BTBD3	-0.397957910160837	0.000103365	0.04241825226597	protein_coding	20
ENSG0000018646	NAP1L2	-0.476759546506481	0.000125339	0.04800015154064	protein_coding	X
ENSG0000011535	TACR1	-0.914107500616397	0.000125923	0.04800015154064	protein_coding	2
ENSG0000000828	CYB561	-0.691717423929374	0.000126993	0.04800015154064	protein_coding	17
ENSG0000008255	OPRK1	-110 152 619 815 561	0.000135605	0.04880501422313	protein_coding	8
ENSG0000017441	TRHR	-18 386 853 595 956	0.000135918	0.04880501422313	protein_coding	8
ENSG0000019812	MB	-202 846 634 247 946	0.000144865	0.05074902525909	protein_coding	22
ENSG0000017445	VWC2L	-137 558 895 103 269	0.000151151	0.05169016702849	protein_coding	2
ENSG0000010315	NECAB2	-0.590564965744378	0.000182785	0.06002485718963	protein_coding	16
ENSG0000016611	SVOP	-0.421858260739423	0.000183881	0.06002485718963	protein_coding	12
ENSG0000015607	WIF1	-100 885 112 895 343	0.000199142	0.06329461211479	protein_coding	12
ENSG0000010009	HPS4	0.200958433374421	0.000202711	0.06329461211479	protein_coding	22
ENSG0000017446	ZCCHC12	-0.48032622203781	0.000228207	0.06753650371389	protein_coding	X
ENSG0000014404	TEX261	-0.400320558381968	0.000229622	0.06753650371389	protein_coding	2
ENSG0000007295	TMEM38A	-0.511541017482356	0.000233619	0.06753650371389	protein_coding	19
ENSG0000007075	PABPC1	0.236901784707127	0.000243889	0.06753650371389	protein_coding	8
ENSG0000013691	WDR38	-307 552 241 631 526	0.000244215	0.06753650371389	protein_coding	9
ENSG0000010578	RUNDC3B	-0.400350680871509	0.000244510	0.06753650371389	protein_coding	7
ENSG0000010143	CST3	-0.486273304553553	0.000253999	0.06883392095285	protein_coding	20
ENSG0000015402	AK5	-0.551564630829809	0.000265025	0.07049200041724	protein_coding	1
ENSG0000002235	GABRA1	-0.868386373104339	0.000275197	0.07094521276154	protein_coding	5
ENSG0000019615	FAT4	0.404024471009851	0.000278066	0.07094521276154	protein_coding	4
ENSG0000010592	ATP6V0A4	335 482 226 858 317	0.000281548	0.07094521276154	protein_coding	7
ENSG0000018418	KCNJ12	-0.708886036383159	0.000319954	0.07743212697807	protein_coding	17
ENSG0000016515	PGAP4	-0.347119734517395	0.000323043	0.07743212697807	protein_coding	9
ENSG0000017787	CCDC184	-0.48086346770791	0.000324567	0.07743212697807	protein_coding	12
ENSG0000005480	CBLN4	-110 073 337 947 398	0.000328856	0.07743212697807	protein_coding	20
ENSG0000019621	ZNF766	0.313098553860097	0.000348930	0.08047682752601	protein_coding	19
ENSG0000010668	LHX2	0.361920845894342	0.000352993	0.08047682752601	protein_coding	9
ENSG0000027288	DCP1A	0.251142262230047	0.000359894	0.08076829334004	protein_coding	3
ENSG0000018213	KCNIP1	-0.717739265319535	0.000384003	0.08485297045474	protein_coding	5
ENSG0000018709	CCK	-150 230 728 769 794	0.000405475	0.08635815123137	protein_coding	3
ENSG0000016895	STXBP6	-0.597710005460222	0.000409553	0.08635815123137	protein_coding	14
ENSG0000017234	RCAN2	-0.620897617553006	0.000410218	0.08635815123137	protein_coding	6
ENSG0000017604	TMPRSS7	-41 596 710 364 357	0.000418462	0.08635815123137	protein_coding	3
ENSG0000016730	MYO5B	0.448595463047258	0.000420877	0.08635815123137	protein_coding	18

ENSG00000165633	VSTM4	-0.837622931898523	0.0004779861	0.09669458507049	protein_coding	10
ENSG00000100427	MLC1	-0.424128651707446	0.0005009171	0.09828164306313	protein_coding	22
ENSG00000196104	SPOCK3	-0.692975098797375	0.0005060727	0.09828164306313	protein_coding	4
ENSG00000126062	TMEM115	-0.292368064519487	0.0005063595	0.09828164306313	protein_coding	3

Supplementary Table 13. DESeq2 DEGs associated to Li treatment in Li-N hCS.

ensembl_gene_id	external_gene_name	log2FoldChange	pvalue	padj	gene_biotype	chromosome
ENSG00000120215	MLANA	228 950 036 679 754	6,84E+01	1,00E+04	protein_coding	9
ENSG00000002822	MAD1L1	-220 450 436 302 945	4,82E+02	3,53E+06	protein_coding	7
ENSG00000119614	VSX2	-212 173 375 558 471	2,86E+03	1,08E+07	protein_coding	14
ENSG00000135373	EHF	-212 348 945 811 129	2,95E+03	1,08E+07	protein_coding	11
ENSG00000118492	ADGB	-210 923 849 649 311	8,27E+03	2,20E+07	protein_coding	6
ENSG00000149452	SLC22A8	-21 049 169 217 603	9,03E+03	2,20E+07	protein_coding	11
ENSG00000284862	CCDC39	-205 790 789 713 924	1,56E+04	3,05E+07	protein_coding	3
ENSG00000163394	CCKAR	-204 111 544 148 032	1,66E+04	3,05E+07	protein_coding	4
ENSG00000177575	CD163	-195 336 127 213 678	1,04E+04	1,70E+08	protein_coding	12
ENSG00000146618	FERD3L	-192 227 415 057 425	1,29E+04	1,88E+08	protein_coding	7
ENSG00000165816	VWA2	-196 599 792 281 815	1,41E+05	1,88E+08	protein_coding	10
ENSG00000095585	BLNK	-196 038 174 212 619	1,57E+05	1,92E+08	protein_coding	10
ENSG00000110777	POU2AF1	-194 130 464 367 819	2,25E+05	2,54E+08	protein_coding	11
ENSG00000175518	UBQLNL	-189 686 816 823 836	5,18E+05	5,42E+08	protein_coding	11
ENSG00000165376	CLDN2	-188 463 050 641 213	6,48E+05	5,93E+08	protein_coding	X
ENSG00000283361	CFAP97D2	-188 463 050 641 213	6,48E+05	5,93E+08	protein_coding	13
ENSG00000134240	HMGCS2	-180 977 222 060 672	2,50E+05	2,16E+09	protein_coding	1
ENSG00000114487	MORC1	171 477 218 227 274	9,62E+06	7,83E+09	protein_coding	3
ENSG00000235718	MFRP	-169 316 677 547 397	1,85E+07	0.0001425629	protein_coding	11
ENSG00000100867	DHRS2	-178 071 322 002 389	2,72E+09	0.0199394027	protein_coding	14
ENSG00000104368	PLAT	224 265 073 204 449	2,97E+09	0.0207160272	protein_coding	8
ENSG00000123119	NECAB1	-11 202 253 300 374	4,03E+09	0.0268283612	protein_coding	8
ENSG00000131095	GFAP	-105 727 956 114 053	4,67E+08	0.0297415183	protein_coding	17
ENSG00000168078	PBK	0.671895697840797	5,10E+08	0.0307627510	protein_coding	8
ENSG00000101335	MYL9	-689 720 409 504 656	5,25E+09	0.0307627510	protein_coding	20
ENSG00000137573	SULF1	-240 887 154 196 444	5,60E+09	0.0315466644	protein_coding	8
ENSG00000164007	CLDN19	-870 770 422 988 925	7,01E+08	0.0380452432	protein_coding	1
ENSG00000006468	ETV1	0.561599375922872	0.00010993837	0.0575095476	protein_coding	7
ENSG00000250120	PCDHA10	19 398 247 530 297	0.000185723207	0.0938030283	protein_coding	5
